# The effect of a single 4CMenB vaccine booster in young people more than ten years after infant immunisation: protocol of an exploratory immunogenicity study

**DOI:** 10.1186/s13063-019-3494-1

**Published:** 2019-07-24

**Authors:** Kimberly Davis, Karen Ford, Rachel Craik, Ushma Galal, Christine S. Rollier, Andrew J. Pollard

**Affiliations:** 1 Oxford Vaccine Group, University of Oxford and the NIHR Oxford Biomedical Research Centre, Oxford, UK; 2 Nuffield Department of Primary Care Health Sciences, University of Oxford, Oxford, UK

**Keywords:** Capsular group B meningococcal vaccination, Capsular group B meningococcal vaccine immunogenicity, Capsular group B meningococcal vaccine childhood schedule, Four-component capsular group B meningococcal vaccine (4CMenB)

## Abstract

**Background and rationale:**

The four-component capsular group B meningococcal vaccine (4CMenB) was introduced into the national immunisation schedule in the UK in September 2015 for infants in a 2 + 1 schedule at two, four and 12 months of age. A two-dose immunisation schedule for adolescents was also considered but was not found to be cost-effective in view of the relatively low rates of disease in this age group. Uncertainty about the longevity of protection induced by the vaccine and lack of certainty about an anamnestic response in primed individuals contributed to this decision.

**Methods/Design:**

This study is an open-label, descriptive immunogenicity analysis. Up to 113 participants will be recruited, including up to 83 children who are now aged 11 years and took part in previous trials involving the administration of 4CMenB to infants, plus a group of 30 naïve age-matched controls. All previously immunised participants will receive one booster dose of 4CMenB. The 30 naïve participants will be randomised to receive two doses of 4CMenB either at 0 and 28 days or 0 and 365 days. Blood samples will be collected from all participants at 0, 28, 180 and 365 days. The primary endpoint will explore immunogenicity at day 0 and 180 in previously immunised and naïve participants. Secondary outcomes will include investigating the persistence of antibody protection in previously immunised participants at the beginning of the study, describing the characteristics of the memory B-cell responses in previously immunised participants, and measuring reactogenicity in all participants following 4CMenB doses.

**Discussion:**

This study aims to describe whether or not a single booster dose of 4CMenB given to those who have received an infant course of 4CMenB induces a recall immune response, while concurrently describing immune responses in naïve children of the same age. If an anamnestic response is proven, a single dose adolescent booster could be envisaged as an addition to the current UK vaccination schedule.

**Trial registration:**

EudraCT, 2017–004732-11. ISRCTN, ISRCTN16774163. Registered on 10 May 2018 (retrospectively registered).

**Electronic supplementary material:**

The online version of this article (10.1186/s13063-019-3494-1) contains supplementary material, which is available to authorized users.

## Background and rationale

Despite the existence of effective antibiotics and recent advances in vaccinology, meningococcal disease remains firmly in the public eye. Of the main *Neisseria meningitidis* capsular groups which cause disease in humans, group B meningococcus (MenB) has the highest incidence in the UK [1] and has proved most difficult to tackle through the design of an effective vaccine. Concerns about a capsular group B meningococcal vaccine based on the polysaccharide capsule inducing poor immunogenicity and possibly eliciting a harmful autoimmune response [2] led to the development of an alternative approach using subcapsular proteins. The four components of 4CMenB include subcapsular antigens and outer-membrane vesicles, specifically a recombinant factor H binding protein (fHbp), *N. meningitidis* capsular group B neisserial heparin binding antigen (NHBA) fusion protein, recombinant *N. meningitidis* group B neisserial adhesin A (NadA) protein, and outer membrane vesicles (OMV) from *N. meningitidis* group B strain NZ98/254 containing PorA P1.4.

4CMenB was licensed in 2013 and introduced into the UK routine vaccination schedule in September 2015 for infants in a 2 + 1 schedule, with the primary series of vaccines at two and four months and a booster dose at 12 months of age. The cost-effectiveness of infant vaccination was borderline, despite this age group suffering the highest rate of disease. Furthermore, an adolescent programme was not initiated in the UK due to the relatively lower rate of disease in this age group, uncertainty over the duration of protection and lack of evidence of induction of wider herd protection [3]. Even when the best-case scenarios of strain coverage (88%), vaccine efficacy (95%) and duration of protection (120 months) as well as the capacity to elicit 30% protection against acquisition of carriage were considered, there was significant uncertainty that an adolescent vaccination programme would be cost-effective at the list price for the vaccine [4].

However, adolescents are responsible for the majority of nasopharyngeal carriage of meningococcus [5] and the majority of transmission. They also suffer from the second highest incidence rate of invasive meningococcal disease (IMD) of any age group [6]. Investing in an adolescent MenB booster programme could further reduce incident cases and may interrupt transmission of hyperinvasive MenB strains, thus reducing overall disease burden.

In 2006, the first infants were vaccinated against MenB as part of 4CMenB clinical development [7]. After three doses of 4CMenB, human serum bactericidal assay (SBA) analysis showed that the vaccine was immunogenic against strains expressing homologous NadA, fHbp and PorA. A recall response was induced after a fourth dose. Some of these children were then recruited to participate in a follow-up study aiming to investigate the persistence of the SBA response and the response induced by a toddler booster injection [8]. The study showed that the booster dose in the 4CMenB-primed participants generated greater increases in SBA titres than in naïve children who were receiving their first ever dose.

These results suggest that a memory B-cell response, induced by 4CMenB received in infancy, was present and supported a recall response two years later. However, the possibility of a strong immune response to 4CMenB vaccination in adolescents more than 10 years after vaccination as infants has not yet been evaluated alongside that of naïve adolescents.

## Methods/Design

### Overview

**Fig. 1:**
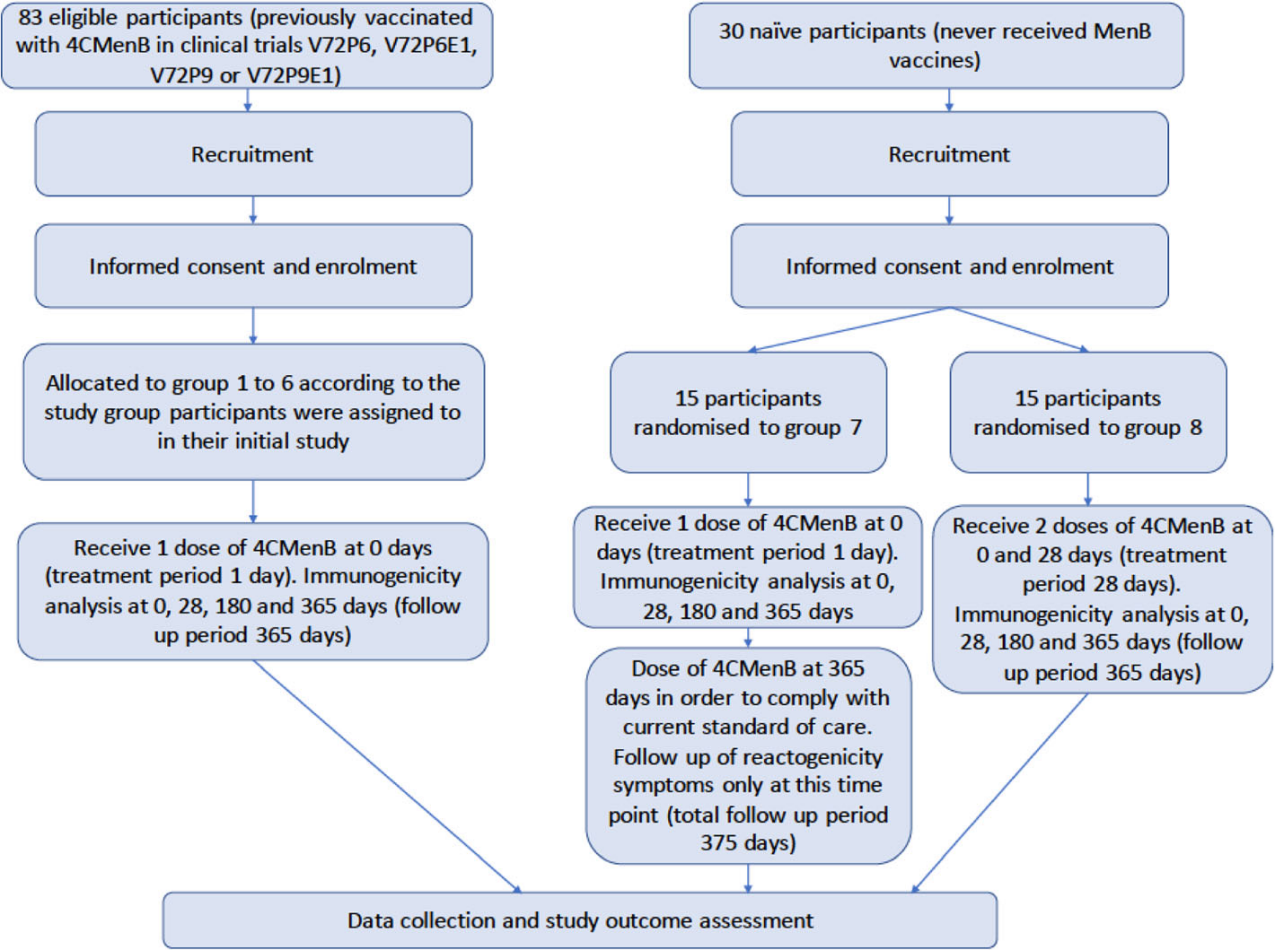
*Flow chart* of the study describing recruitment, group allocation/randomisation, treatment and follow-up

This is an unblinded exploratory immunogenicity analysis. Six participant groups will be allocated and two participant groups will be randomised (Fig. 1). This study has been approved by the Nottingham 2 Research Ethics Committee (reference 17/EM/0466) and is registered with the EudraCT clinical trials database (2017–004732-11) and the ISRCTN database (ISRCTN16774163). The study will be conducted in accordance with the Declaration of Helsinki.

See Table 1 for further details on the schedule of 4CMenB that participants in groups 1 to 6 received in the initial study or studies they took part in.

**Table 1:** Previous schedule of 4CMenB received by groups 1–6

Study Group	Number of subjects	Initial study number and group	Past vaccination with 4CMenB
1	13	V72P6 Group 4	12 months
2	13	V72P9 Group 2	6, 8, and 12 months
3	8	V72P6E1 Group 4	12 months and boost 40 and 42 months
4	12	V72P9E1 Group 2	6, 8, and 12 months and boost 40 months
5	22	V72P6 Group 2	2, 4, 6 and 12 months
6	19	V72P6E1 Group 2	2, 4, 6 and 12 months and boost 40 months

### Recruitment

Recruitment will take different approaches depending upon whether participants have been previously vaccinated.

Previous trial participants who had consented to being contacted regarding future vaccine research will be informed of the study by invitation letter. They will also receive parent and child study information booklets sent via email or stamped addressed envelope. Contact details provided in previous studies and stored on a database will be used to contact these participants. If it is not possible to contact participants, the investigators will request local Child Health Information Services (CHIS) forward on study information to the current listed address for these potential participants.

Naïve participants will be informed of the study through website-based advertising, social media and poster advertisements. Families registered with the Oxford Vaccine Group (OVG) research database will also be contacted. Study information booklets may be disseminated via GP practices, in educational/recreational settings, in schools and school newsletters. The investigators may also request mail outs or emails from CHIS.

### Participants and enrolment

As this is a preliminary study to explore the persistence and potential impact of antigen-specific memory B-cell responses on an adolescent 4CMenB vaccination programme, all statistical analyses will be descriptive; therefore, a formal sample size calculation was not carried out and the decision of the sample size in groups 7 and 8 was purely pragmatic. The sample size of participants in groups 1–6 is determined by the number of participants who completed the previous studies. It is anticipated that approximately 50% of the 83 previously immunised participants may return to take part in this study. This would give a best-case scenario of 113 total participants, with overall participant numbers likely closer to 71 (half of the previously immunised participants and 30 naïve participants).

A trained study staff member, either a nurse or doctor, will enrol participants. Parents/legal guardians will be required to sign a consent form and participants will be required to sign an assent form, both of which will be retained by investigators.

Samples from this study would be eligible for an ancillary study entitled BioBank (Oxford Vaccine Centre Biobank 2009/09, Oxford Vaccine Centre Biobank Ethics Ref: 16/SC/0141). Separate consent/assent is sought for this. Taking part in Biobank is optional.

### Inclusion criteria

Separate inclusion criteria apply to those participants who were previously vaccinated as opposed to naïve participants. Groups 1–6 will consist of children who have previously taken part in clinical trials V72P6 [7], V72P6E1 [8], V72P9 [9] or V72P9E1 [10]. Groups 7 and 8 will be children with no major medical conditions, whose date of birth falls within the same six-month period as the children in groups 1–6 (25 June 2006 to 17 December 2006) and who have not previously received any MenB vaccines.

### Exclusion criteria

There are general exclusion criteria for all groups and exclusion criteria specific to whether participants have been previously vaccinated.

Exclusion criteria for all groups include those whose parents are on the delegation log for the study, that have a history of invasive MenB disease or a household contact with a confirmed case of meningococcal meningitis. Participants should not have confirmed or suspected immunodeficiency, a family history of congenital or hereditary immunodeficiency, or maternal HIV. Participants should not have a history of an anaphylactic reaction to any component of 4CMenB. They should not have any other significant disease or disorder which, in the opinion of the investigator, may either put them at risk because of participation in the trial, or may influence the result of the trial, or the participant’s ability to participate in the trial. Participants should not have participated in another research trial involving an investigational product in the 12 weeks prior to taking part in this study, and they should not be planning to receive any other investigational vaccine or drug. They should not have thrombocytopenia or any other bleeding disorder and should not have received blood, blood products or plasma derivatives within the past 3 months.

#### Exclusion criteria specific to groups 1–6

Participants in these groups should not have received any previous vaccination with 4CMenB except as part of V72P6, V72P6E1, V72P9 or V72P9E1 clinical trials and they should not have had any other MenB vaccine. They should not have received immunosuppressive therapy (anti-cancer chemotherapy or radiation therapy) within the preceding month or long-term systemic corticosteroid therapy within the last month (e.g. oral prednisolone > 0.5 mg/kg/day or intravenous glucocorticoid steroid). Nasal, topical or inhaled steroids are allowed.

#### Exclusion criteria specific to groups 7 and 8

Participants in these groups should not have received previous vaccination with any MenB vaccine. They should not have ever received immunosuppressive therapy (anti-cancer chemotherapy or radiation therapy). They should not currently be receiving long-term systemic corticosteroid therapy (e.g. oral prednisolone > 0.5 mg/kg/day or intravenous glucocorticoid steroid). Nasal, topical or inhaled steroids are allowed. They should not be receiving long-term prophylactic antibiotics.

### Randomisation

Participants in study groups 1–6 will not be randomised. They will receive the same treatment, as detailed above.

Naïve participants will be randomised to groups 7 or 8 with an allocation ratio of 1 : 1. This will be achieved by the use of a block randomisation list generated in STATA/IC 14.2. The randomisation will be implemented using sequentially numbered, opaque, sealed paper envelopes which will be kept in a locked cupboard until the first study visit during which the naïve participant is randomised. The allocation sequence has been generated by the study statistician at the Nuffield Department of Primary Care Health Sciences, University of Oxford. Clinical study staff will take the next numbered randomisation envelope to the first study visit. After enrolment the clinical staff member will open the randomisation envelope and unblind the clinical staff and study participant.

### Interventions

**Fig. 2:**
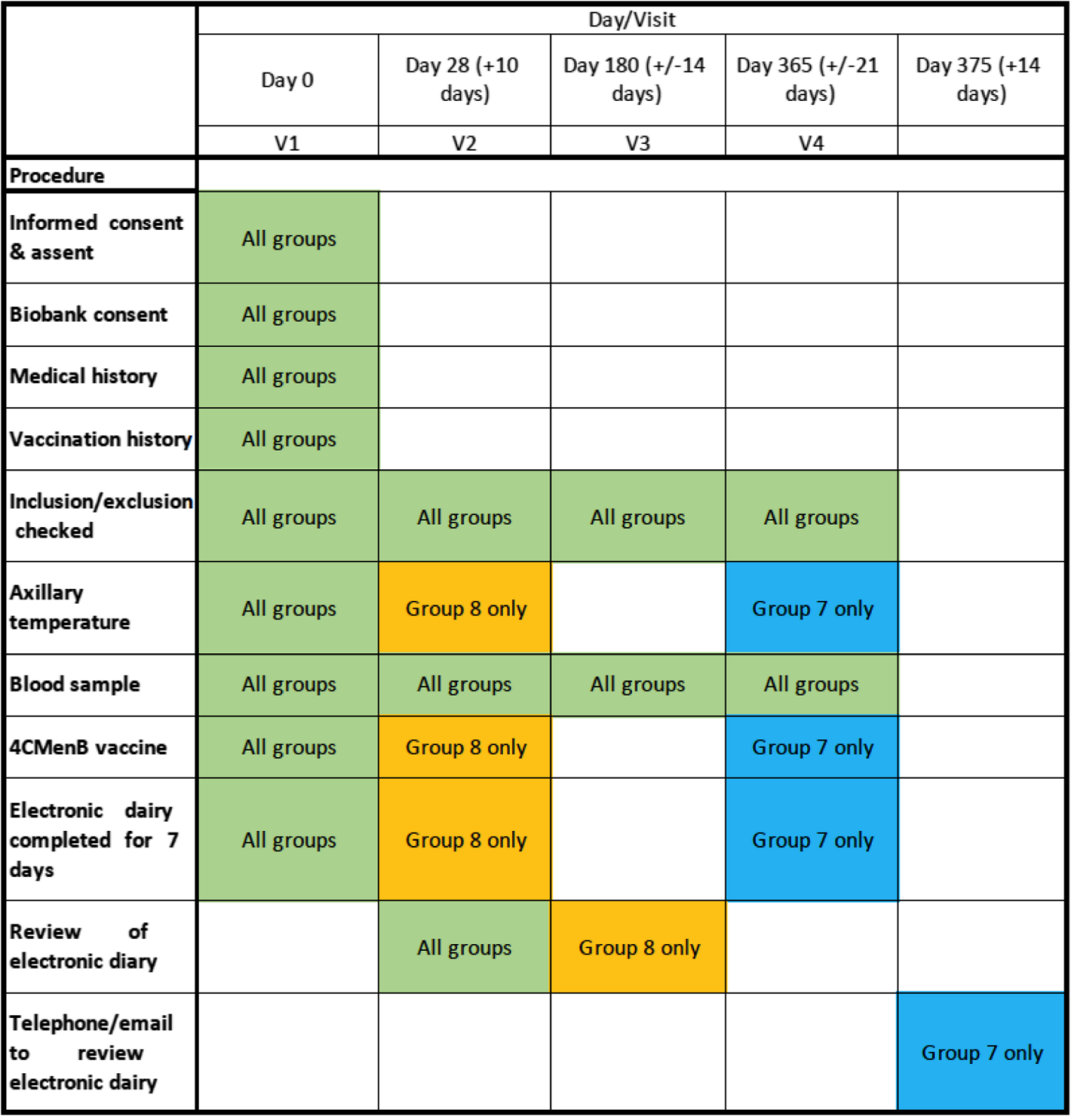
Participant timeline

All participants will receive 4CMenB 0.5 mL intramuscularly according to product specifications [11] following the timeline in Fig. 2. Vaccines and venipuncture will be done by trained study nurses or doctors. Study visits will take place in clinics at the local tertiary level hospital (John Radcliffe Hospital, Oxford, UK) or in the homes of participants across Oxfordshire, Berkshire, Buckinghamshire and neighbouring counties in the south-west UK.

### Adverse events

Inclusion and exclusion criteria are checked before each study visit. If a participant no longer fulfils all of the inclusion and none of the exclusion criteria, they would automatically be disqualified from continuing in the study. Participants who have an adverse reaction that they find unacceptable would be able to withdraw at any time. Participants suffering from possible anaphylaxis to 4CMenB would be withdrawn from the study.

### Laboratory methods

#### Primary outcome

The ability of antibodies in participants’ serum samples to mediate killing of meningococci in the presence of complement (hSBA) will be quantified. The target strains in the hSBA assay will be H44/76, 5/99 and NZ98/254. The hSBA titre will be calculated as the lowest concentration of the serum dilution giving a 50% reduction of colony-forming units (CFUs) of a specified inoculum of bacteria after incubation with participant serum in the presence of complement.

#### Secondary outcomes

Analyses to quantify the B- and T-cell responses specific to 4CMenB may be performed when feasible using peripheral blood mononuclear cells (PBMCs) and plasma derived from study participants sampled before and after each vaccine dose, using the assays described below. If possible, additional hSBA will also be performed against a panel of wild-type UK and EU circulating strains.

B-cell responses will be analysed by the ability of 4CMenB to stimulate a detectable increase in IgG, IgA and IgM-producing antigen-specific memory B-cells enumerated by ELISPOT using plates coated with vaccine antigens (OMVs, fHbp, NHBA, NadA and/or PorA). The phenotype and kinetics of the B-cell subsets involved in the response can be determined by flow cytometry. In addition, other assays to monitor the B-cell immune response to the vaccines may be performed if sufficient samples are available.

In order to explore the ability of 4CMenB to stimulate T-cell responses, we aim to quantify (when possible) vaccine-induced responding T-cells by proliferation or production of cytokines upon stimulation of PBMCs with meningococcal vaccine antigens after immunisation, as compared to pre-immunisation (by ELISPOT or intra-cellular cytokine assays). The phenotype of the responding effector and memory T-cell subsets can be characterised by flow cytometry and intra-cellular cytokine assay when possible. Moreover, other assays to monitor the T-cell immune response and cytokine release to the vaccines may be performed if sufficient samples are available.

Binding of serum IgG and IgM antibodies to human factor H (FH) may be measured by ELISA, performed as described in previous studies [12]. If serum anti-FH reactivity is detected, recombinant specific human FH domains can be used to determine the location of the FH epitopes reactive with the antibody. If detected, further exploratory assays will be performed to characterise the function and persistence of anti-FH antibody.

### Statistical methods and study outcomes

#### Statistical methods

As mentioned above, the number of participants vaccinated as infants in previous trials is limited. Due to small sample sizes, the statistical analysis will be purely descriptive and results will be reported in the form of percentages, frequencies, geometric mean titres and geometric mean fold rises with 95% confidence intervals (CI), per group and per strain (for three strains: H44/76, 5/99, NZ98/254). For geometric mean titres and fold rises, data will be log transformed (base 10) and CIs will be calculated on this log scale, before transforming back to the original scale for reporting and interpretation. CIs for proportions will be calculated using the binomial exact method. No statistical tests will be carried out.

### Primary objective

A descriptive analysis will be performed after all participants have completed visit 3 (day 180) in order to evaluate the primary endpoint of the study. This will be done by calculating the geometric mean of fold change in hSBA titre from day 0 to day 180, for each group for which these data are available.

### Secondary objectives (for the six follow-on groups only)

At day 0, the persistence of the protective antibody response will be summarised in adolescents who received infant vaccination as well as in adolescents who received infant vaccination with toddler boosting. The percentage of participants with an hSBA titre ≥ 1 : 4; the geometric mean of hSBA titres will be calculated and presented together with 95% CIs.

### Exploratory objectives (all groups)

At days 0, 28, 180 and 365 the percentage of participants with an hSBA titre ≥ 1 : 4 and the geometric mean of hSBA titres will be calculated together with 95% CIs. For B-cell memory and other immune cell types, summary statistics (mean (SD) or median (IQR), as determined by the distribution of the data) will be presented.

### Reactogenicity analysis

The frequency and severity of local and systemic solicited vaccine reactions will be summarised separately for each treatment group as frequencies and percentages for each dose of 4CMenB.

### Methods for any additional analyses

The principal descriptive analysis will be performed on a per-protocol basis where the population for the immunogenicity analysis will consist of all participants who receive one or two doses of the vaccine (depending on the group) and provide at least one evaluable serum sample (after or before vaccination). An intention-to-treat analysis will also be performed in which all participants will be included in the analysis if they are successfully vaccinated on day 0. If a participant later withdraws from the study, data up until that point will be included in the analysis.

## Discussion

The UK was the first country in the world to introduce 4CMenB into its routine immunisation schedule, which has successfully reduced IMD in infants, but not in other age groups [13]. There has been a general public acceptance of 4CMenB, with 88.6% of eligible infants receiving the two recommended doses of vaccine in the first 10 months of it being made publicly available [14]; there are strong calls from the UK public to include 4CMenB in the routine immunisation schedule for older children [15]. Children who took part in previous UK-based 4CMenB trials as infants are now approaching adolescence; therefore, there is a unique opportunity to specifically address several uncertainties about 4CMenB immunisation in adolescents with important public health implications.

The persistence of immune responses to 4CMenB was recently evaluated 4 and 7.5 years after the initial two doses of vaccine [16]. Participants in this trial were older than this study (age range 15–24 years) and those that were previously vaccinated received priming doses of vaccine in childhood or adolescence, not infancy. The trial showed that previously immunised participants had higher baseline antibody levels than naïve controls (for all vaccine antigens except NHBA), as well as a stronger immune response post booster vaccination, suggesting antibody persistence and an anamnestic response. 4CMenB has also already been evaluated in naïve adolescents. A Chilean study demonstrated robust immune responses against vaccine antigens after two doses, with only a small, non-significant increase in immunogenicity achieved after a third dose of vaccine [17]. Other studies in different populations in Canada and Australia [18] and the United States [19] have confirmed a protective immune response in adolescents after two doses of vaccine. In a US study of an outbreak on a college campus, no further cases of MenB disease occurred in those that had received at least one dose of 4CMenB, indicating that even one dose of vaccine may be protective. 4CMenB has also been shown to be generally well tolerated in adolescents with no safety concerns identified [16–18, 20].

This study will add to the established literature by describing whether there is a persistent protective antibody response and B-cell memory in adolescents eight to 10 years after infant vaccination or infant vaccination with toddler boosting, and if the protective antibody response to an adolescent booster among individuals immunised in early childhood is higher and/or more persistent than in naïve adolescents. While the level of vaccine-induced protective antibody titres against MenB approximately 10 years after the last vaccination is expected to be low, previously immunised adolescents may have persistent vaccine-specific memory B-cells. This may in turn produce a strong antibody recall response to a new encounter with the antigen in the form of a booster vaccine. The information arising from this study will complement UK-based adolescent carriage studies that are currently being carried out by the Department of Health [21], as well as other international studies [22].

There are some weaknesses of this study, including the fact that the number of participants that can be recruited from previous studies is fixed. Around half the number of previous trial participants are expected to return, given that it has been approximately 10 years since the original study finished. They may be difficult to locate or simply not wish to take part. While this restricted sample size means that there is not sufficient power to demonstrate substantial differences, this important observational study could inform future investigation and hypothesis generation in the area. The study is unblinded for participants and clinical staff, but is blinded for lab staff; therefore, the immunogenicity analysis has a low risk of bias.

Participant follow up from this study is expected to be completed by March 2020 (24-month study period). This will be one of the first studies to describe possible anamnestic immune responses to 4CMenB after infant priming alongside the immune response generated following immunisation of naïve participants. The data will provide new evidence to inform the potential utility of adolescent booster programmes for 4CMenB, which may be of interest in policy development (Additional file 1).

## Trial status

Protocol version 4.0 dated 21 June 2018.

Date of first enrolment: 24 March 2018.

Approximate date of recruitment completion: March 2019.

Approximate date of trial completion: March 2020 (24-month trial period).1

## Additional file

Additional file 1: SPIRIT checklist. (DOCX 3509 kb)

## Data Availability

The datasets used and/or analysed during the current study are available from the corresponding author on reasonable request.

## Abbreviations

4CMenB: Four component meningococcal B vaccine (Bexsero®)
CHIS: Child Health Information Services
ELISA: Enzyme-linked immunosorbent assay
ELISPOT: Enzyme-linked immunosorbent spot assay
EU: European Union
FH: Human factor H
fHbp: Factor H binding protein
HIV: Human immunodeficiency virus
hSBA: Human serum bactericidal antibody
IMD: Invasive meningococcal disease
MenB: Meningococcal capsular group B
NadA: Neisserial adhesin A
NHBA: Neisserial heparin binding antigen
NHS: National Health Service UK
NIHR: National Institute of Health Research
OVG: Oxford Vaccine Group
PBMC: Peripheral blood mononuclear cell
PorA: Neisserial outer membrane protein
