# Supplementary material for: The effect of a single 4CMenB vaccine booster in young people more than ten years after infant immunisation: protocol of an exploratory immunogenicity study
Source: Trials. 2019 Jul 24;20:455. doi: 10.1186/s13063-019-3494-1 (PMC6657159; doi:10.1186/s13063-019-3494-1)
Supplement: Supplementary file 1 — SPIRIT checklist. (DOCX 3509 kb) [file 13063_2019_3494_MOESM1_ESM.docx]

**Preventing meningococcal disease in young people after infant immunisation: effect of a single 4CMenB vaccine booster over 10 years of age. Protocol of an exploratory immunogenicity study.**

**SPIRIT Checklist:**

**Administrative information:**

**1) Title:**

Preventing meningitis in young people after infant immunisation: effect of a single meningococcal 4CMenB vaccine booster over 10 years of age.

**2a) Trial registration:**

EudraCT: 2017-004732-11

ISRCTN 16774163

**2b) Trial registration Data Set:**

1. Primary registry and trial identifying number: ISRCTN 16774163

2. Date of registration in primary registry: 10^th^ May 2018

3. Secondary identifying numbers: EudraCT: 2017-004732-11

4. Sources of monetary support: Meningitis Research Foundation

NHS National Institute of Health Research

Oxford Biomedical Research Centre.

5. Primary sponsor: University of Oxford

6. Secondary sponsor: N/A

7. Contact for public enquiries: Professor Andrew Pollard

Oxford Vaccine Group

Churchill Hospital

Oxford OX3 7LE

+441865 611400

[Andrew.pollard@paediatrics.ox.ac.uk](mailto:Andrew.pollard@paediatrics.ox.ac.uk)

8. Contact for scientific enquiries: Professor Andrew Pollard

Oxford Vaccine Group

Churchill Hospital

Oxford OX3 7LE

+441865 611400

[Andrew.pollard@paediatrics.ox.ac.uk](mailto:Andrew.pollard@paediatrics.ox.ac.uk)

9. Public title: Meningococcal B Booster vaccine in young

people.

10: Scientific title: Preventing meningitis in young people after

infant immunisation: effect of a single

meningococcal 4CMenB vaccine booster over

10 years of age.

11. Countries of recruitment: United Kingdom

12. Health condition(s) or problem(s) studied: Capsular group B meningococcal vaccination

Capsular group B meningococcal vaccine immunogenicity

13. Intervention(s):

Intervention name: 4CMenB (Bexsero^®^ –GSK, Rixensart, Belgium)

Intervention description:

Previously immunised children:

Group 1-6: 4CMenB given on day 0. Blood tests at Day 0, 28, 180 and 365.

Naïve children:

Group 7: 4CMenB given on day 0 and 365. Blood tests at Day 0, 28, 180 and 365.

Group 8: 4CMenB given on day 0 and 28. Blood tests at Day 0, 28, 180 and 365.

14. Key inclusion and exclusion criteria:

Inclusion criteria:

For recruitment to study groups 1 to 6 only

- Healthy children aged approximately 11 years who have completed a vaccination course of 4CMenB as an infant or toddler in clinical trials V72P6, V72P6E1, V72P9 or V72P9E1.

For recruitment to Naïve groups 7 and 8 only

- Healthy children aged approximately 11 years who have not previously received 4CMenB vaccine.

Exclusion criteria:

All groups

- History of invasive meningococcal B disease.
- History of being a household contact with a case of confirmed meningococcal meningitis
- Confirmed or suspected immunodeficiency.
- A family history of congenital or hereditary immunodeficiency, or maternal HIV.
- History of anaphylactic reaction to any component of the vaccine.
- Any other significant disease or disorder which, in the opinion of the Investigator, may either put the participants at risk because of participation in the trial, or may influence the result of the trial, or the participant’s ability to participate in the trial.
- Thrombocytopenia or any bleeding disorder.
- Receipt of blood, blood products, or plasma derivatives within the past 3 months.

Exclusion to study groups 1 to 6 only

- Any previous vaccination with 4CMenB or another MenB vaccine except as part of V72P6, V72P6E1, V72P9 or V72P9E1 clinical trials.
- Receipt of immunosuppressive therapy such as anti-cancer chemotherapy or radiation therapy within the preceding 1 month or long-term systemic corticosteroid therapy.

Exclusion to Naïve groups 7 and 8 only

- Previous vaccination with 4CMenB vaccine or with any other meningococcal B vaccine.
- Receipt of immunosuppressive therapy such as anti-cancer chemotherapy or radiation therapy.
- Current receipt of long-term systemic corticosteroid therapy.
- Long term prophylactic antibiotic use.

15. Study type: An open-label, exploratory immunogenicity study.

16. Date of first enrolment: 24^th^ March 2018

17: Sample size: Target = 113 participants; 83 previously immunised

(anticipated 50% enrolment in this group) and 30 naïve.

Currently enrolled = 40 previously immunised, 12 naïve.

18. Recruitment status: Actively recruiting.

19. Primary outcome:

Serum bactericidal activity against meningococcal strains at day 0 and 180 days post-immunisation, measured by validated assay.

20. Key secondary outcomes:

- Serum bactericidal activity before vaccination, measured at day 0.
- Serum bactericidal activity against meningococcal strains. Quantification and qualitative characterisation of B-cell memory and other immune cell types, measured at day 0, 28, 180 and 365 post-immunisation.
- Parental recording of eDiary information including axillary temperature check at 6 hours post vaccination. Reactogenicity data gathered for 7 days after any vaccines given.

21: Ethics review:

This trial has been approved by Nottingham 2 Research Ethics Committee (reference 17/EM/0466).

22: Completion date: Expected completion date March 2020 (24 month trial

period).

23: Summary results: Not yet available.

24: IPD sharing statement:

The results, interpretation and conclusions will be disseminated to parents and participants via letter and will be submitted for publication in peer-reviewed journals. The findings of this study could also be used to inform vaccination policy in the U.K.

**3) Protocol version:**

Protocol version 4.0 dated 21^st^ June 2018

Revision chronology

**Original:** Protocol version 1.0 dated 20^th^ November 2017.

**First amendment:** Protocol version 2.0 dated 30^th^ January 2018. Addition of the option to return a reply slip for previously vaccinated participants when indicating whether they would like to take part in this study. Changing the randomisation of participants in the naïve group from a computer-based system (Sortition) to a paper envelope-based system to avoid issues with internet access within participants’ homes.

**Second amendment:** Protocol version 3.0 dated 19^th^ March 2018. Amendment of the protocol and parent and assenting information booklets to change the use of a continuous temperature monitoring system to being optional. Addition of reply slips for the general practitioner (GP) and Child Health Information Service (CHIS) so that they can notify us if they have managed to forward an information pack to a family. Amended typographical errors in group card 7 and 8, study labels and the assenting information booklet for non-naives.

**Third amendment:** Protocol version 4.0 dated 21^st^ June 2018. Changing of the statistical analysis section to include a descriptive analysis of immunogenicity at 180 days, and changes to the wording of how the analysis will be done. Changing of the wording of the exclusion criteria from “household contact of bacterial meningitis” to specify “meningococcal meningitis”. Updated the patient information leaflet to change the details of where to opt-out of receiving information on future trials via mail and to update according to the General Data Protection Regulation (GDPR).

**4) Funding:**

The study is part funded through a research grant from the Meningitis Research Foundation (MRF), project 1702.0 (study groups 5 to 8) and part funded through a research grant from the NHS National Institute of Health Research Oxford Biomedical Research Centre.

**5a) Roles and responsibilities:**

**Protocol contributors:**

DAVIS Kimberly^1^ is the lead doctor and assisted with protocol drafting and finalisation as well as study delivery.

FORD Karen^1^ is the lead nurse, and has led on protocol drafting, development of study documents, participant recruitment and delivery of the study.

CRAIK Rachel^1^ was the project manager and led on seeking regulatory approvals, as well as study document development.

GALAL Ushma^2^ is a statistician who provided advice regarding statistical analysis of the primary and secondary endpoints.

ROLLIER Christine^1^ led on design of the study, funding applications, and provided laboratory expertise with regards to protocol development.

POLLARD Andrew^1^ conceived of the study idea, approved the final protocol and is Principal Investigator.

^1^Oxford Vaccine Group, Centre for Clinical Vaccinology and Tropical Medicine, University of Oxford and the NIHR Oxford Biomedical Research Centre.

^2^Nuffield Department of Primary Care Health Sciences, University of Oxford.

**5b) Sponsor contact information:**

University of Oxford

Joint Research Office
Boundary Brook House

Churchill Drive

Headington

Oxford, OX3 7LQ

Ph: 01865 572245

Fax: 01865 572228

Email: [ctrg@admin.ox.ac.uk](mailto:ctrg@admin.ox.ac.uk)

**5c) Role of sponsor and funders:**

The University of Oxford provided guidance regarding study design, but play no role regarding the collection, management, analysis and interpretation of data.

MRF and NHS National Institute of Health Research Oxford Biomedical Research Centre play no role in the design and implementation of this project.

**5d) Overseeing groups:**

The University of Oxford Trials Safety Group (TSG) will conduct a review of all SAEs for the trial reported during the quarter and cumulatively. The aims of this committee include:

- To pick up any trends, such as increases in un/expected events, and take appropriate action.
- To seek additional advice or information from investigators where required.
- To evaluate the risk of the trial continuing and take appropriate action where necessary.

**Introduction:**

**6a) Background and rationale:**

Despite the existence of effective antibiotics and recent advances in vaccinology, meningococcal disease remains firmly in the public eye. Of the main *Neisseria meningitidis* capsular groups which cause disease in humans, group B meningococcus (MenB) has the highest incidence in the U.K.(1) and has proved most difficult to tackle through the design of an effective vaccine. Concerns about a capsular group B meningococal vaccine based on the polysaccharide capsule inducing poor immunogenicity and possibly eliciting a harmful autoimmune response(2) led to the development of an alternative approach using subcapsular proteins. The four components of 4CMenB consist of subcapsular antigens and outer-membrane vesicles, including a recombinant factor H binding protein (fHbp), *Neisseria meningitidis capsular* group B neisserial heparin binding antigen (NHBA) fusion protein, recombinant *Neisseria meningitidis* group B neisserial adhesin A (NadA) protein and outer membrane vesicles (OMV) from *Neisseria meningitidis* group B strain NZ98/254 containing PorA P1.4.

4CMenB was licensed in 2013 and introduced into the UK routine vaccination schedule in September 2015 for infants in a 2+1 schedule (primary series at two and four months, booster dose at 12 months of age). The cost-effectiveness of infant vaccination was borderline, despite this age group suffering the highest rate of disease. Furthermore, an adolescent programme was not initiated in the UK due to the relatively lower rate of disease in this age group and uncertainty over the duration of protection in this age group and the potential for induction of wider herd protection(3). Even when the best-case scenarios of strain coverage (88%), vaccine efficacy (95%), duration of protection (120 months), and the capacity to elicit 30% protection against acquisition of carriage were considered, an adolescent programme was estimated to not be cost-effective at the list price for the vaccine.

However, adolescents are responsible for the majority of nasopharyngeal carriage of meningococcus(4) and the majority of transmission. They also suffer from the second highest incidence rate of invasive meningococcal disease (IMD) of any age group(5). Investing in an adolescent MenB booster programme will not only reduce incident cases, but also may interrupt transmission of hyperinvasive MenB strains, thus reducing overall disease burden.

In 2006, the first infants were vaccinated against MenB in a trial in Oxford as part of 4CMenB clinical development(6) and received 4CMenB at two, four, six, and 12 months of age. After three doses, human serum bactericidal assay (SBA) analysis showed that 4CMenB was immunogenic against strains expressing homologous NadA, fHbp and PorA. A recall response was induced after the fourth dose at 12 months of age. Some of these children were then recruited to participate in a follow-up study, aiming to investigate the persistence of the SBA response, and the response induced by a toddler booster injection given at 40 months of age(7). This showed that the booster dose in the 4CMenB-primed participants generated greater increases in SBA titers than in naïve children who were receiving their first ever dose at 40 months of age. These results suggest that a memory B-cell response, induced by 4CMenB received in infancy, was present and supported a recall response two years later. From the children who took part in the first study (vaccinations received under 12 months of age), some received a boost at 40 months and some did not.

4CMenB has also been evaluated in naïve adolescents, and it was demonstrated that two doses induced robust immune responses against the vaccine antigens, including bactericidal activity (SBA)(8-10). The vaccine was well tolerated, and no safety concerns were identified(11). 4CMenB is currently licensed for adolescent use, with naïve adolescents requiring two injections at a minimum 1-month interval. However, a possible booster immune response in adolescents after infant vaccination with 4CMenB has not yet been evaluated or compared with that of naïve adolescents.

While the level of vaccine-induced protective antibody titres against MenB more than ten years after the last vaccination is expected to be low, previously immunised adolescents may have a substantial level of vaccine-specific memory B-cells. This may in turn produce a strong antibody recall response to a new encounter with the antigen in the form of a booster vaccine. If this was the case, a single dose adolescent booster could be envisaged as an addition to the current vaccination schedule. Information that will arise from U.K.-based adolescent carriage studies that are currently being carried out by the Department of Health will also help to inform this decision.

**7) Objectives:**

Children who took part in previous 4CMenB trials are now approaching adolescence, and therefore we are presented with the unique possibility to specifically address the following questions:

1. What is the persistence of the protective antibody response and B-cell memory in adolescents 10 years after infant vaccination?
2. What is the persistence of protective antibody responses and B-cell memory among adolescents after infant vaccination with toddler boosting (eight years after the last 4CMenB vaccination)?
3. Is the protective antibody response to an adolescent booster among individuals immunised in early childhood higher and/or more persistent than in naïve adolescents?

Analysis of bactericidal activity (primary outcome).

The ability of the antibodies in participants’ serum samples to mediate killing of meningococci in the presence of complement (serum bactericidal activity (SBA)), will be quantified. The target strains in the SBA assay will be a panel of wild-type strains, in order to elucidate the antigen-specific SBA titre. The SBA titre will be calculated as the lowest concentration of the serum dilution giving a 50% reduction of colony forming units (CFUs) of a specified inoculum of bacteria after incubation with participant serum in the presence of complement.

Cellular responses and cytokine release (exploratory outcome).

Laboratory analyses to quantify the B- and T-cell responses specific to 4CMenB may be performed when feasible using peripheral blood mononuclear cells (PBMCs) and plasma derived from study participants sampled before, and after each vaccine dose, using the assays described below:

- Analyses of B cell responses: The ability of 4CMenB to stimulate a detectable increase in IgG, IgA and IgM producing antigen-specific memory B cells enumerated by ELISPOT using plates coated with vaccine antigens (OMVs, fHbp, NHBA, NadA and/or PorA). The phenotype and kinetics of the B-cell subsets involved in the response can be determined by flow cytometry. In addition, other assays to monitor the B-cell immune response to the vaccines may be performed if sufficient samples are available.

- Analyses of T cell responses and cytokine release: In order to explore the ability of 4CMenB to stimulate T cell responses, we aim to quantify (when possible) vaccine-induced responding T-cells by proliferation or production of cytokines upon stimulation of PBMCs with meningococcal vaccine antigens after immunisation, as compared to pre- immunisation (by ELISPOT or intra-cellular cytokine assays). The phenotype of the responding effector and memory T-cells subsets can be characterised by flow cytometry and intra-cellular cytokine assay when possible. Moreover, other assays to monitor the T-cell immune response and cytokine release to the vaccines may be performed if sufficient samples are available.

- Serum antibody reactivity with human factor H (FH): Binding of serum IgG and IgM antibodies to human FH may be measured by ELISA, performed as described in previous studies(12). If serum anti-FH reactivity is detected, recombinant specific human FH domains can be used to determine the location of the FH epitopes reactive with the antibody. If detected, further exploratory assays will be performed to characterise the function and persistence anti-FH antibody.

**8) Trial design:**

An open-label exploratory immunogenicity analysis.

**SPIRIT figure for study timeline:**

**
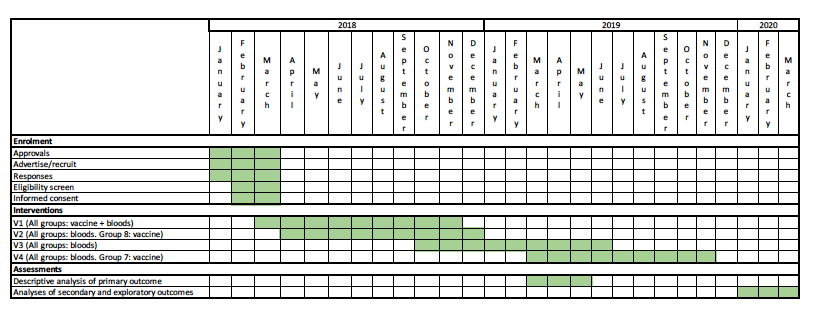
**

**9) Study setting:**

Study visits will take place in clinics at the local tertiary level hospital, or in the homes of participants. The site will depend upon several factors including participant preference and distance from the study centre. A list of study sites is below:

- John Radcliffe Hospital, Oxford.
- Participant homes across Oxfordshire, Berkshire, Buckinghamshire and neighbouring counties.

**10) Eligibility criteria:**

Key inclusion and exclusion criteria:

Inclusion Criteria for recruitment to all study groups:

- Parents willing and able to comply with trial protocol requirements.
- Parents have given informed consent.
- Participant is willing and able to give informed assent.

For recruitment to study groups 1 to 6 only

- Healthy children who have taken part in clinical trials V72P6, V72P6E1, V72P9 or V72P9E1.

For recruitment to Naïve groups 7 and 8 only

- Healthy children age-matched with groups 1-6 who have not previously received 4CMenB.

Exclusion Criteria

- Children of parents who are on the delegation log for this study.
- History of invasive meningococcal B disease.
- Household contact with a case of confirmed meningococcal meningitis.
- Confirmed or suspected immunodeficiency.
- A family history of congenital or hereditary immunodeficiency, or maternal HIV.
- History of anaphylactic reaction to any component of the vaccine.
- Any other significant disease or disorder which, in the opinion of the Investigator, may either put the participants at risk because of participation in the trial, or may influence the result of the trial, or the participant’s ability to participate in the trial.
- Participants who have participated in another research trial involving an investigational product in the past 12 weeks.
- Prior or planned receipt of any other investigational vaccine or drug.
- Thrombocytopenia or any bleeding disorder.
- Receipt of blood, blood products, or plasma derivatives within the past 3 months.

Exclusion to study groups 1 to 6 only

- Any previous vaccination with 4CMenB vaccine except as part of V72P6, V72P6E1, V72P9 or V72P9E1 clinical trials.
- Any previous vaccination with another meningococcal B vaccine.
- Receipt of immunosuppressive therapy (anti-cancer chemotherapy or radiation therapy) within the preceding 1 month or long-term systemic corticosteroid therapy (e.g. oral prednisolone >0.5ml/kg/day or intravenous glucocorticoid steroid). Nasal, topical or inhaled steroids are allowed.

Exclusion to Naïve groups 7 and 8 only

- Previous vaccination with 4CMenB vaccine or with any other meningococcal B vaccine.
- Receipt of immunosuppressive therapy (anti-cancer chemotherapy or radiation therapy).
- Current receipt of long-term systemic corticosteroid therapy (e.g. oral prednisolone >0.5ml/kg/day or intravenous glucocorticoid steroid). Nasal, topical or inhaled steroids are allowed.
- Long term prophylactic antibiotic use.

**11a) Interventions for each group:**

All participants will be required to undergo four episodes of venepuncture as part of the immunogenicity analysis. All naïve participants (groups 7 and 8) will receive two doses of 4CMenB either on day 0 and 28 or day 0 and 365. All follow-on participants (groups 1-6) will receive one dose of 4CMenB on day 0.

**11b) Criteria for discontinuing or modifying allocated interventions:**

N/A

**11c) Strategies to improve adherence to intervention protocols:**

Strategies include home visits and the use of anaesthetic cream prior to venepuncture.

**11d) Relevant concomitant care and interventions permitted:**

The only restrictions are listed in the exclusion criteria.

**12) Outcomes:**

Primary outcome:

Serum bactericidal activity against meningococcal strains at day 0 and 180 days post-immunisation, measured by validated assay.

Key secondary outcomes:

- Serum bactericidal activity before vaccination, measured at day 0.
- Serum bactericidal activity against meningococcal strains. Quantification and qualitative characterisation of B-cell memory and other immune cell types, measured at day 0, 28, 180 and 365 post-immunisation.
- Parental recording of eDiary information for 7 days post vaccination. Reactogenicity data gathered at all time points after vaccines given.

**13) Participant timeline:**


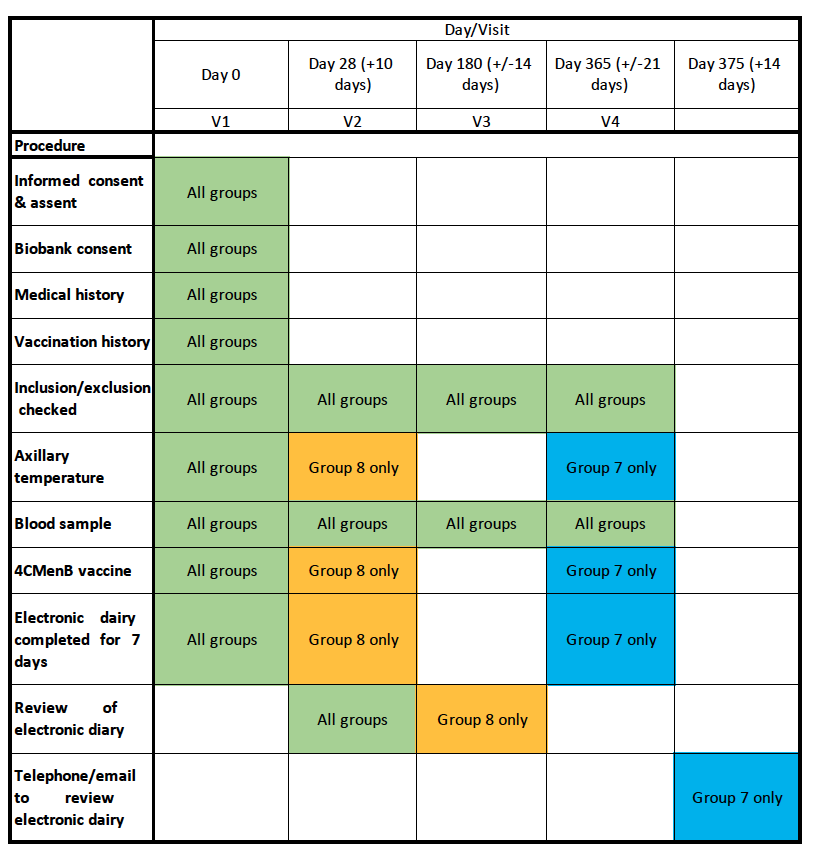


**14) Sample size:**

This is a preliminary study to explore the persistence and potential impact of antigen-specific memory B cell responses on an adolescent programme, therefore a formal sample size calculation was not carried out and the decision of the sample size in groups 7 and 8 was purely pragmatic. The sample size of the participants in groups 1 to 6 is determined by the number of participants who completed the previous studies in 2006-2009 at Oxford. We anticipate approximately 50% of the 83 previously immunised participants to return to take part in the upcoming study however we aim to recruit as many as possible to improve statistical power.

**15) Recruitment:**

Recruitment, approach and initial eligibility assessment of potential study participants:

Recruitment will take place in geographical areas where appropriate approvals have been granted.

Recruitment to groups 1 to 6 only (previously vaccinated)

Parents/legal guardians of potential participants who previously consented to be contacted regarding future vaccine research will be informed of the study by mailing out and/or emailing invitation letters with the parent and child study information booklets, reply slip, and stamped, addressed envelope. The contact details provided to the Oxford Vaccine Group while participating in clinical trials V72P6, V72P6E1, V72P9 or V72P9E1 will be used.

Recruitment to groups 7 and 8 only (naïve)

Parents/legal guardians of potential participants will be informed of the study through website-based advertising, social media, contacting families registered with the OVG research database, and poster advertisements. Dissemination of the study information booklets, including at GP practices, in educational/recreational settings, mail outs/emails from Child Health Information Service or schools and school newsletters may also be employed.

**16a) Allocation sequence generation:**

Participants in study groups 1 to 6 will not be randomised. The study group to which they are assigned is determined by the previous vaccination course of 4CMenB they received while participating in studies V72P6, V72P6E1, V72P9 or V72P9E1, and they will all receive the same study interventions at the same time points.

Naïve group participants will be randomised to Groups 7 or 8 with an allocation ratio of 1:1. This will be achieved by the use of a randomisation list generated in STATA/IC 14.2.

**16b) Allocation concealment mechanism:**

The randomisation will be implemented using sequentially numbered, opaque, sealed paper envelopes which will be kept in a locked cupboard until the first study visit during which the naïve participant is randomised.

**16c) Implementation:**

The allocation sequence has been generated by the study statistician at the Nuffield Department of Primary Care Health Sciences, University of Oxford. Clinical study staff take the next numbered randomisation envelope to the first study visit. After enrolment the clinical staff member will open the randomisation envelope and unblind the staff and study participant.

**17a) Blinding:**

This study is unblinded after allocation of groups 7 and 8.

**17b) Circumstances permitting unblinding:**

N/A

**18a) Data collection methods:**

Standardised paper data collection forms are completed by dedicated, trained study staff at each visit. The data in these forms are then transcribed into OpenClinica, a computer-based data collection software package. Blood samples are collected by staff specifically trained in paediatric phlebotomy. The volume of blood collected and the site of venepuncture are recorded. The site of the vaccine administration is also recorded.

Parents are asked to complete an online or paper diary detailing reactogenicity for seven days following vaccine administration.

**18b) Plans to promote retention and complete follow up:**

Participants attending clinic will be given a small reimbursement for their travel costs and parking. Participants who receive home visits will not receive payments. Participants who miss clinic study visits will be offered a follow up visit at home if practicable.

**19) Data management:**

In this study, the paper CRF and eDiary entries will be considered source data. Data for analysis will be entered onto an electronic database or eCRF (OpenClinica or REDCap (Research Electronic Data Capture)). Other documents such as medical notes and GP letters may be used as source documents if required.

Access to Data

Direct access will be granted to authorised representatives from the Sponsor, host institution and the regulatory authorities to permit trial-related monitoring, audits and inspections.

Data Recording and Record Keeping

All study files with demographic and clinical details on the participants will be kept in a locked research office at Centre for Clinical Vaccinology and Tropical Medicine (CCVTM). The study data will be subsequently entered on to a computer with an electronic database protected by a password and encrypted transfer. All stored blood samples will be identified by study number only and will have no personal identifiers.

Information on study participants will be recorded on hard copy source documents held locally, and information will be entered into a web based electronic CRF (eCRF, OpenClinica™ or REDCap database stored on a secure server within the UK and has restricted access and is password-protected with accountability records).

**20a) Statistical methods:**

We have been limited in this study due to the number of participants vaccinated in previous trials as infants. Due to small sample sizes, the statistics will be descriptive, reported in the form of percentages, frequencies, geometric mean titers and geometric mean fold rises with 95% confidence intervals, per group and per strain (for the three strains: H44/76, 5/99, NZ98/254). For geometric mean titers and fold rises, data will be log transformed (base 10), and confidence intervals will be calculated on this log scale, prior to transforming back onto the original scale for reporting and interpretation. Confidence intervals for proportions will be calculated using the binomial exact method.

Primary objective

A descriptive analysis will be performed after all participants have completed visit 3 (day 180) in order to evaluate the primary endpoint of the study. This will be done by calculating the geometric mean of fold change in hSBA titre from Day 0 to Day 180, for each group for which this data is available.

Secondary objectives (For the six follow-on groups only)

To assess at Day 0 the persistence of the protective antibody response in adolescents after infant vaccination compared to adolescents after infant vaccination with toddler boosting, the following statistical parameters and their 95% confidence intervals will be determined as outlined above:

• Percentage of participants with an hSBA titre ≥ 1:4

• Geometric mean of hSBA titres

Exploratory objectives

At Days 0, 28, 180 and 365 post-treatment:

- the following statistical parameters and their 95% confidence intervals will be determined for each of the previously immunised groups, as outlined above:
  - Percentage of participants with an hSBA titre ≥ 1:4
  - Geometric mean of hSBA titres
- For B-cell memory and other immune cell types, summary statistics (Mean (SD) or Median (IQR), as determined by the distribution of the data) will be presented

Reactogenicity Analysis:

The frequency and severity of local and systemic solicited vaccine reactions will be summarised as frequencies and percentages for the participants overall for each dose of 4CMenB vaccine, separately for each treatment group.

**20b) Methods for any additional analyses:**

The principal comparisons will be performed on a per-protocol basis where the population for the immunogenicity analysis will consist of all participants who receive one or two doses of the vaccine (depending on the group) and providing at least one evaluable serum sample (after or before vaccination). An intention-to-treat analysis will also be performed where all participants will be included in the analysis if they are successfully vaccinated on Day 0. If a participant later withdraws from the study, data up until that point will be included in the analysis.

**20c) How to deal with protocol non-adherence or missing data:**

All available data will be used in the analysis except that which is from participants excluded from the per-protocol populations. Missing data will not be imputed.

**21a) Data monitoring:**

Regular monitoring will be performed by CTRG (Clinical Trials and Research Governance), the study monitor, according to GCP.

The P.I. will submit (in addition to the expedited reporting above) DSURs once a year throughout the clinical trial, or on request, to the Competent Authority (MHRA in the U.K.), Ethics Committee, HRA (where required) and Sponsor.

**21b) Description of interim analyses and stopping guidelines:**

A descriptive analysis will be performed after all participants have completed visit 3 (day 180) in order to evaluate the primary endpoint of the study.

The Principal Investigator and Trial Safety Group will have the right to recommend termination of the study at any time on grounds of participant safety. If the study is prematurely terminated the investigator will promptly inform the participants. If the study is halted, the MHRA and relevant Ethics Committee will be notified within 15 days of this occurring.

**22) Harms:**

The Medicines for Human Use (Clinical Trials) Regulations contain a requirement for the notification of "serious breaches" to the MHRA within 7 days of the Sponsor becoming aware of the breach.

In the event that a serious breach is suspected the Sponsor must be contacted within 1 working day. In collaboration with the P.I., the serious breach will be reviewed by the Sponsor and, if appropriate, the Sponsor will report it to the REC committee, Regulatory authority within seven calendar days.

**23) Auditing:**

N/A

**24) Research ethics approval:**

This trial has been approved by Nottingham 2 Research Ethics Committee (reference 17/EM/0466).

**25) Protocol amendments:**

Protocol amendments will be communicated to relevant parties via email or phone call as necessary.

**26a) Consent or assent:**

A written version and verbal explanation of the study information leaflet and informed consent will be presented to the parent/legal guardian of the participant and the participant detailing:

- the exact nature of the trial.
- the implications and constraints of the protocol.
- the known side effects and any risks involved in taking part.
- Sample handling – participants will be informed that anonymised samples taken during the course of study may be shared with study collaborators, including collaborators outside of the European Union, for the purposes of this study.

It will be clearly stated that the participant is free to withdraw from the trial at any time for any reason without prejudice to future care, without affecting their legal rights and with no obligation to give the reason for withdrawal.

Written informed consent/assent will be obtained by means of dated signatures of both the parent/participant and of the person who presented and obtained the informed consent/assent. The person who obtained the consent must be suitably qualified and experienced and have been authorised to do so by the Principal Investigator. A copy of the signed informed consent/assent forms will be given to the parent. The original signed forms will be retained at the trial site.

**26b) Ancillary studies:**

The parent/legal guardian of the participant will also be informed that their samples would be eligible for BioBank (Oxford Vaccine Centre Biobank 2009/09, Oxford Vaccine Centre Biobank Ethics Ref: 16/SC/0141)**.** Separate consent/assent is sought for this. Biobank is optional.

**27) Confidentiality:**

The trial staff will ensure that the participants’ anonymity is maintained. The participants will be identified only by a participant ID number on all trial and source documents and any electronic database, with the exception of the paper CRF, where participant initials may be added. All documents will be stored securely and only accessible by trial staff and authorised personnel. The trial will comply with the GDPR, which requires data to be anonymised as soon as it is practical to do so.

**28) Declaration of competing interests:**

AJP reports grants from Okairos, and Pfizer, which finished within the past 36 months outside the submitted work. AJP is Chair of UK Dept. Health’s Joint Committee on Vaccination & Immunisation & the EMA scientific advisory group, on vaccines and is a member of the WHO’s SAGE.

**29) Access to data:**

Direct access will be granted to authorised representatives from the Sponsor, host institution and the regulatory authorities to permit trial-related monitoring, audits and inspections.

**30) Ancillary and post-trial care:**

There will not be provision of 4CMenB vaccine beyond the trial period.

The University of Oxford has a specialist insurance policy in place which would operate in the event of any participant suffering harm as a result of their involvement in the research (Newline Underwriting Management Ltd, at Lloyd’s of London).

**31a) Dissemination policy:**

The Investigators will co-ordinate dissemination of data from this study. All publications (e.g., manuscripts, abstracts, oral/slide presentations, book chapters) based on this study will be reviewed by each sub-investigator prior to submission. Authors will acknowledge that the study was funded by the Meningitis Research Foundation and NIHR Oxford Biomedical Research Centre. Trial participants will be notified of the results of the study upon publication of the study.

**31b) Authorship eligibility and use of professional writers**

Authorship will be determined in accordance with the ICMJE (International Committee of Medical Journal Editors) guidelines and other contributors will be acknowledged. There is no intention to utilise professional writers.

**31c) Plans for granting public access to the full protocol, participant-level dataset, and statistical code**

The datasets used and/or analysed during the current study are available from the corresponding author on reasonable request.

**Appendices:**

**32) Informed consent materials:**


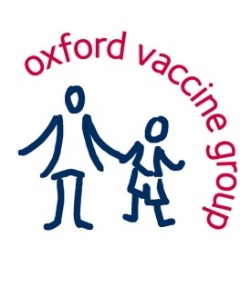


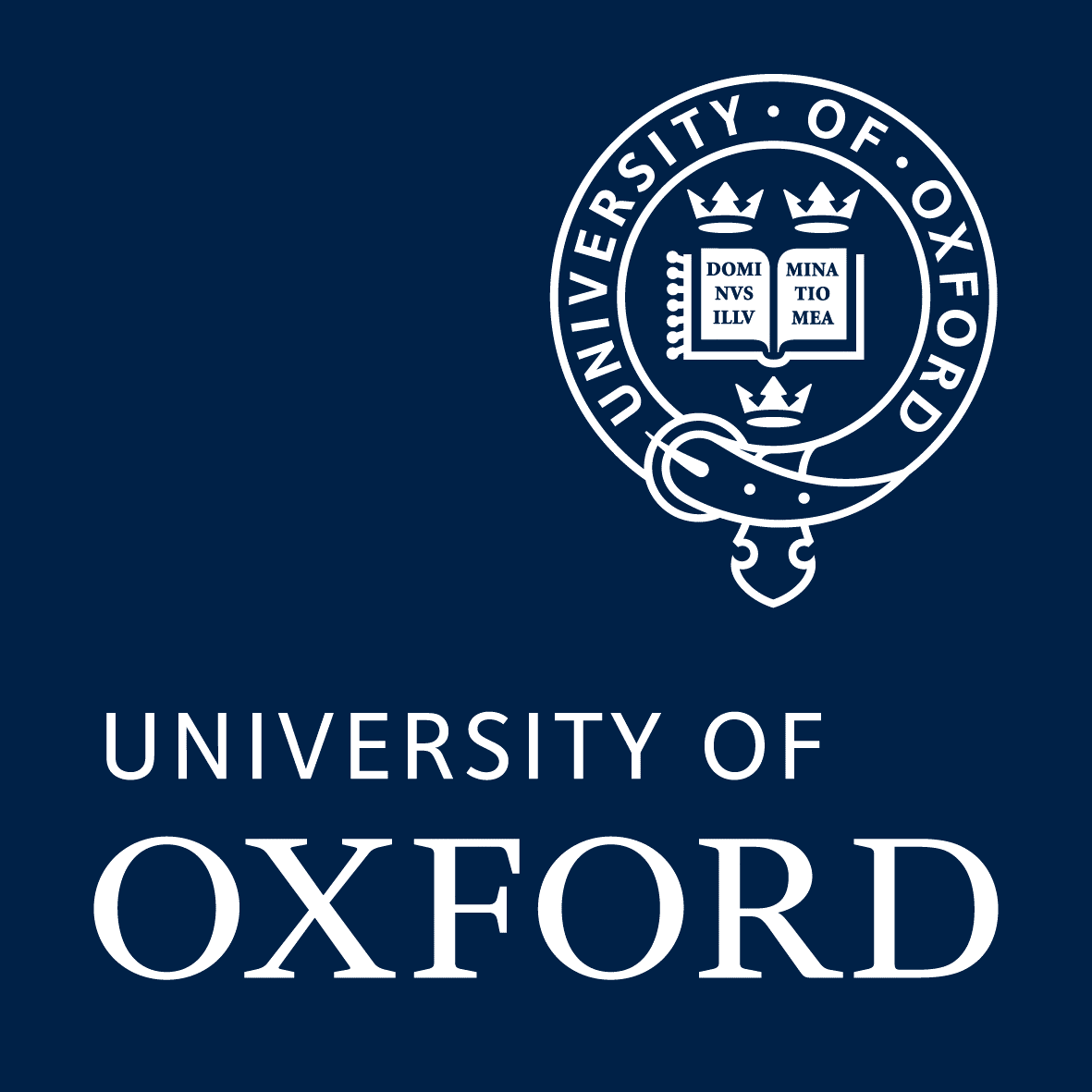


OXFORD VACCINE GROUP

| Meningococcal B Booster Vaccine in Young People |
| --- |

**Parent/Legal Guardian Information Booklet**

Children are at risk of meningitis and septicaemia (blood poisoning) due to a bacteria called meningococcus. Understanding how young people respond to vaccination against this disease may help to protect them in the future**.**

Oxford Vaccine Group (OVG) are inviting your child to take part in a

a study looking at the responses that young people make to a licensed vaccine (4CMenB) against meningococcus group B (MenB). We are recruiting 2 groups of childen: those who received the vaccine as an infant or toddler; and a group of children who did not.

Children who received the 4CMenB vaccine as an infant or toddler form a unique and special group. We want to assess how well the vaccine they received in the past is still protecting them from MenB , especially if we give them a booster vaccine, compared with children who have never receieved the vaccine.

Before you decide whether you would like your child to take part you should understand what the study is about and what participation would involve. Please read the information carefully and discuss with your child and others if you wish. There is an separate information leaflet available designed for your child to read. If anything is unclear, or you would like further information, please contact the study team.

Thank you for considering taking part in this study.

**Contact Details:** Oxford Vaccine Group, Centre for Clinical Vaccinology and Tropical Medicine (CCVTM) Churchill Hospital Oxford OX3 7LE

**Tel:** 01865 611400 **Fax** 01865 289695 Email: [**info@ovg.ox.ac.uk**](mailto:info@ovg.ox.ac.uk)

### **Table of contents Page**

| **Who are the Oxford Vaccine Group?** | 3 |
| --- | --- |
| Why has my child been invited to take part? | 3 |
| Why are vaccines important? | 4 |
| What is meningococcal infection? | 4 |
| **What is this study about?** | 4 |
| What happens in this study? | 5 |
| Where will the study visits happen? | 5 |
| How will the blood test be taken? | 6 |
| How will the 4CMenB vaccines be given? | 6 |
| What will we ask you to do after each vaccine? | 6 |
| Table summaring what happens in this study | 7 |
| What are the possible side-effects of the vaccines? | 7 |
| What are the possible side-effects of blood sampling? | 8 |
| **Does my child have to take part in the study?** | 8 |
| **What are the benefits of taking part?** | 8 |
| **What will happen to the samples obtained in the study?** | 9 |
| **Would my child’s taking part in this study be kept confidential?** | 9 |
| **Who else can see my child’s study records?** | **9** |
| **What will happen if I don’t want my child to carry on with the study?** | **10** |
|  |  |
|  |  |
| **What will happen at the end of the research study?** | **10** |
| **What if I wish to complain?** | **10** |
| **What else do I need to know?** | **11** |
| So, in summary, what would happen if I decide to take part in the study? | **11** |
| **What should I do now if I’m interested in taking part?** | **12** |

Dear Parent/Legal Guardian,

The Oxford Vaccine Group (OVG) would like to invite your child to be involved in a study assessing how young people respond to a vaccine against meningococcus group B (MenB). Approval for this study has been gained from the East Midlands – Nottingham 2 Research Ethics Committee.

### **Who are the Oxford Vaccine Group?**

The Oxford Vaccine Group, which is part of the University of Oxford, is an independent research team of Doctors, Nurses and Play Assistants. We carry out research studies on topics related to infectious diseases and vaccines for children and adults. In the past 5 years alone over 7,000 participants in the Thames Valley area have taken part in our research studies.

**Why has my child been invited to take part?**

We are inviting two groups of children who are aged about 11 years to be involved in the study:

1. A unique group of children who took part in an Oxford Vaccine Group study when they were an infant/toddler in which they received 4CMenB, the vaccination against meningococcus group B (MenB)
2. Healthy children who did not receive a MenB vaccine as an infant/toddler, who are within the age range for this study and living in an area where this study is being carried out

If your child previously took part in an Oxford Vaccine Group study and you agreed to hear about future research, we have used the contact details that you gave to us at that time, or asked your GP to send this invitation.

If your child did not did receive MenB vaccine as an infant/toddler, this invitation has either been posted to you by the National Health Applications and Infrastructure Services (NHAIS) who hold the central NHS patient database, via your child’s school or your GP surgery. Please note that the Oxford Vaccine Group has not been given your child’s name and address.

### Why are vaccines important?

Vaccines stimulate our immune system to protect us from infectious diseases by making us produce something called antibodies in our blood. This is an immune response. If a child comes into contact with an infectious disease against which they have been vaccinated (or “immunised”), the antibodies will help their body to recognise and fight the disease. Without vaccines children are at increased risk of catching many serious diseases.

### What is meningococcal infection?

Meningococcal infection is caused by a family/group of bacteria (or germs) called meningococcus. Meningococci are carried naturally in the back of the throat (or nasopharynx) and are most commonly found in adolescents. They don’t usually cause any harm. However, in some situations the bacteria can overcome the body’s defence and cause meningitis (infection around the surface of the brain) or septicaemia (blood poisoning). This is rare, but can be life-threatening, and mostly affects babies, young children and adolescents.

There are 5 main groups of the meningococcus family that cause disease: A, B, C, W and Y. In the UK, from the beginning of July 2015 to the end of June 2016 there were 805 confirmed cases of meningococcal disease; 15% occurred in 15-24 years olds. MenB accounted for 55% of all cases; or 444 out of the 805 cases.

**What is this study about?**

In this study we are seeking to understand the immune response of young people to the 4CMenB vaccine, the vaccine against MenB infection. We are trying to determine if children who received the vaccine as infants and toddlers are still protected from MenB infection as they enter adolescence, and if they have a stronger immune response with just a single booster (“top up”) vaccine than those who have never received the vaccine.

4CMenB has been given to infants and toddlers as part of the routine immunisation schedule in the U.K. since September 2015. It has not been given to adolescents as part of the schedule due to concerns about cost-effectiveness. If the vaccine can be given as a one-off booster to those that received the vaccine as infants, rather than two doses that are currently required in adolescents who never previously received the vaccine, then this could be a cost-effective and acceptable way to protect adolescents from MenB infection.

### What happens in this study?

Children will receive one or two doses of the licensed 4CMenB (Bexsero®) vaccine and have four blood tests taken over the course of a year.

Children who received a course of 4CMenB vaccine as an infant or toddler whilst taking part in an Oxford Vaccine Group research study (the same vaccine that is now used in the routine infant immunisation schedule) are being invited to take part if their parents agreed to hear about future research studies. All previously vaccinated children will receive one dose of 4CMenB.

In addition a group of about 30 children of the same age, around 11 years (born between 25/06/2006 and 17/12/2006), who have never received any 4CMenB vaccine are being recruited to this study (they will make up the control groups). These children will receive two doses of 4CMenB. The children in the control groups will be randomised, like flipping a coin, to receive two doses of 4CMenB vaccine either one month or one year apart. You or the study team would not be able to influence which vaccine schedule your child is randomised to receive.

To test the immune response to the 4CMenB vaccine, all children who take part in the study will have four blood tests performed, spaced out over a year, as shown in the table below. The blood samples taken before receiving a 4CMenB vaccine in this study will allow us to see if there is an immune response still present after being vaccinated as a baby or toddler. Samples collected from the control groups will provide a comparison.

Blood samples collected at visits 2, 3 and 4 are taken after all children have received one dose of 4CMenB vaccine in this study. This will allow us to compare the immune response between one dose of 4CMenB in those vaccinated as an infant or toddler and those who were not.

### Where will the study visits happen?

There will be a total of 4 visits for all children. All visits and study procedures would be conducted at your home or within a central location such a hospital outpatients clinic. Study visits would take place before or after school, at weekends or during school holidays and all but the first would take around 30 minutes. The total duration of the study for each child will be 12 months.

At the first visit you and your child would be given the chance to discuss the study in more detail, including the risks and benefits of participation. If you were happy for your child to take part we would ask you to sign the consent form and you would be given a copy of this to keep. We would ask your child to sign an assent form. One of our study doctors or nurses would ask you some questions about your child’s medical history to ensure he/she was able to be included. This visit may take longer than the others, approximately 1 hour.

### How will the blood test be taken?

The amount of blood taken at each visit will be 20ml, approximately four teaspoons. This will be taken from the inner arm in the elbow crease or the back of the hand. In order to minimise discomfort from these blood tests we would use an anaesthetic cream or cold spray to help numb the skin. At the first visit the OVG staff would put it on and for all the other blood test visits, we would give you the cream and instructions to put it on before study visits.

### How will the 4CMenB vaccines be given?

The 4CMenB vaccine would be given by injection into the top of your child’s non-dominant arm.

**What will we ask you to do after each vaccine?**

After the vaccination visits, you would need to measure and record your child’s temperature as well as any side effects from the vaccine that he/she may have.

We would ask you to record within an electronic diary (e-Diary) how your child was feeling in the seven days after receiving the vaccine. In addition, we would require you to record details of any illnesses requiring a medical visit and any medicines given to treat these for the 28 days following vaccination.

Children in groups 1-6, which differ depending on their previous vaccination schedule, will only need to complete the e-Diary once because they are only receiving one dose of vaccine. Children in group 8 will have a second dose of vaccine at visit 2 (four weeks after the first dose) and will be asked to complete the e-Diary for 28 days for a second time. Children in group 7 who receive their second vaccine at the final study visit (visit four, one year after the first dose) are only required to complete the e-Diary for seven days after this final study visit.

Recording any vaccine side effects is a very important part of the study. As this will be done by using an electronic diary (e-Diary), having access to the internet during the study period is crucial. The e-Diary will allow us to capture both expected side-effects (e.g. fever, redness, swelling and pain around the injection site) and non-expected side effects from the vaccines. Completing the e-Diary is usually quick and you will be shown how to use it by a Doctor or Nurse.

After each 4CMenB vaccine we may also ask you to monitor your child’s temperature continuously for 24 hours. We would issue you with a special temperature monitoring device for this as well as instructions on how to fit it, and what to do with it at the end of the monitoring period. It would be about the size of a small watch and would be worn around the wrist. It needs to be removed for activities like bathing, showering and swimming and then put on again afterwards.

You will be provided with a telephone number to enable you to have 24-hour access to one of our paediatric doctors for telephone advice should you have any concerns about your child’s health following vaccination.

### Table summaring what happens in this study

| **Study Group** | **Visit 1** Day 0 | **Visit 2** Day 28 (one month) | **Visit** 3 Day 180  (six months) | **Visit 4** Day 365 (one year) |
| --- | --- | --- | --- | --- |
| **1- 6** | **Blood sample**  **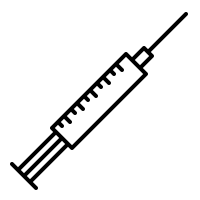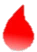 + Vaccine**  **Diary**  **Temperature monitoring** | **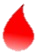Blood sample** | **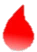Blood sample** | **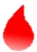Blood sample** |
| **7** | **Blood sample**  **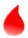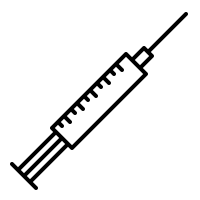+ Vaccine**  **Diary**  **Temperature monitoring** | **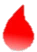Blood sample** | **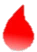Blood sample** | **Blood sample**  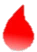**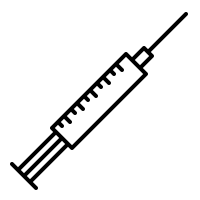+ Vaccine**  **Diary**  **Temperature monitoring** |
| **8** | **Blood sample**  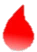**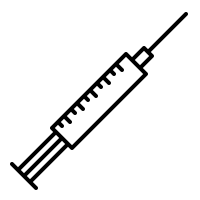+ Vaccine**  **Diary**  **Temperature monitoring** | **Blood sample**  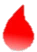**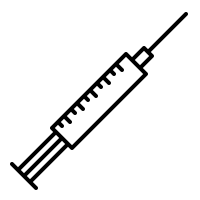+ Vaccine**  **Diary**  **Temperature monitoring** | **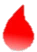Blood sample** | **Blood sample**  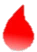 |

### What are the possible side-effects of the vaccines?

The 4CMenB vaccine is a licensed vaccine, meaning that it has met the rigorous safety standards required by European medicine safety organisations.

Your child may have some side effects from immunisation with the 4CMenB vaccine. 4CMenB is not a live vaccine and therefore cannot cause a meningitis infection. Side effects that we would expect to see after immunisation generally are pain, swelling or redness at the site of the injection and more general effects such as fever, malaise (general feeling of being unwell), muscle pain, irritability and headache. With 4CMenB vaccine, the most common side effects in young people (from 11 years of age) and adults were pain at the injection site, malaise and headache. Paracetamol or Ibuprofen can be given to relieve these reactions if they occur.

In addition to the reactions listed above, there may be reactions to the 4CMenB vaccine that are not yet known because it has never been given as a booster dose to those that have been immunised as infants or toddlers. However, as the 4CMenB vaccine is part of the UK national infant immunisation schedule, and recommended for all individauls with increased risk factors due to certain medical conditions, its safety is continually being monitored through the routine safety surveillance systems.

As with all vaccines, there is a small chance of an allergic reaction to the 4CMenB vaccine so we would monitor your child for 15 minutes following vaccination. The study nurses/doctors are specifically trained and equipped to deal with this unlikely event.

### What are the possible side-effects of blood sampling?

There may be some pain at the blood sampling site, as well as possible bleeding and/or bruising following the blood sample.

### **Does my child have to take part in the study?**

No, taking part in research is voluntary. If you decided not to participate this would not affect your child’s routine care in any way. You are free to change your mind at any point in the study. Whatever you choose it is important that you and your child are happy with your decision and it is not the role of the study team to help decide for you.

### **What are the benefits of taking part?**

We anticipate that the 4CMenB vaccine will protect against most types of MenB disease. Although it is already licensed, 4CMenB is not currently routinely given to young people in the UK. Information from this study will help us learn more about how the vaccine could been used in the future as a booster (topping up) to extend protection against meningococcal group B disease.

Please note that if your child is randomly allocated to group 7 then the final vaccine at Visit 4 is to complete the recommended vaccination schedule for adolescents.

### **What will happen to the samples obtained in the study?**

Blood samples obtained in the study would be labelled with your child’s study code and number but not his/her name. They will be stored in a freezer at the Oxford Vaccine Group until the analysis takes place. The samples may be shipped to collaborators outside of the UK and the European Union, for analysis.

Separately, we will ask you for permission to store left over components of your child’s blood, including DNA (genetic material), in a collection of samples called a ‘BioBank’. Details of this will be provided to you before you are enrolled into this 4CMenB vaccine study. You are free to say no to the BioBank study and continue to take part in this 4CMenB study.

At the completion of the study any remaining blood samples will be destroyed if you have not given consent for these to be retained in the Oxford Vaccine Centre Biobank.

### **Would my child’s taking part in this study be kept confidential?**

We inform your child’s GP after you and your child have consented to taking part in the this study. We will also let them know when we have given the 4CMenB vaccine.

The University of Oxford is the data controller for this study, and is therefore responsible for looking after your information and using it properly. All personal information and samples collected from your child will be coded with a study number and kept strictly confidential. Your child’s information would be stored on a secure server hosted by the University of Oxford and paper notes would be held by the Oxford Vaccine Group in a locked filing cabinet. Only authorised study staff can access your child’s data and samples. Your child’s personal information (name, date of birth, and contact information) is kept separately to their study results and will only be used to contact you about the study or for medical reasons. Following completion of the study all study records (which includes some personal data such as name, date of birth and contact details) will be retained up to 3 years after the youngest participant reaches 18 years of age. We would also seek your permission to use the data in future related research. Files will be confidentially destroyed when no longer required. Your rights to access, change or move your information are limited as we need to manage your information in specific ways in order for the research to be reliable and accurate. To safeguard your rights, we will use the minimum personally identifiable information possible. You can find out more about how we use your information by contacting the study team.**Who else can see my child’s study records?**

In order to ensure that the study is being conducted correctly the study records can be inspected by the Clinical Trials and Research Governance Office (CTRG), University of Oxford, without violating yours child’s confidentiality. This group is responsible for ensuring the appropriate conduct and accuracy of the research on behalf of the research sponsor (University of Oxford).

By signing the consent form for this study you would be giving permission for CTRG to look at your child’s medical records, however they would not be able to remove information that identified your child from the Oxford Vaccine Group premises.

Your child’s study information, removed of any identifying information, might also be used for additional medical and/or scientific research projects in the future. If you do not want the information used in this way, or have any questions about the use of your child’s information in the study, please inform the study team.

### **What will happen if I don’t want my child to carry on with the study?**

You can change your mind and withdraw your child from the study at any time without giving any reason. If you change your mind and withdraw your child from the study we would use the samples and data we have collected up until the point you informed us that you wanted to withdraw, unless you inform us in writing that you wish for your child’s data and samples to be destroyed.

### **What will happen at the end of the research study?**

The results of the research will be published in a scientific medical journal; this potentially can take up to 2 years. All Oxford Vaccine Group publications will appear on the Oxford Vaccine Group website and you will receive a letter containing these results. Your child would not be identified in any report or publication and we will not provide individual results.

If you are interested in hearing about other research studies that we may be running in the future then there is an option to sign up to an Oxford Vacine Group Children and Young People’s Database through which we can get in touch. This does not oblige you in any way to take part in the future research.

### **What if I wish to complain?**

If you wish to complain about any aspect of the way you have been approached or treated during the course of this study, you should contact the Oxford Vaccine Group on 01865 611400 or email [info@ovg.ox.ac.uk](mailto:info@ovg.ox.ac.uk).

You can also contact the University of Oxford Clinical Trials and Research Governance (CTRG) office on 01865 572224 or email the Head of CTRG Heather House ctrg@admin.ox.ac.uk.

If you do not wish to receive invitations of this kind in the future, please register your child on the Oxford Vaccine Group opt-out list at www.trials.ovg.ox.ac.uk/trials/opt-out.

The University of Oxford, as Sponsor, has appropriate insurance in place in the unlikely event that your child suffers any harm as a direct consequence of participating in this study. Similarly, the venues where the study may be conducted will also have appropriate insurance; more information can be provided as required.

**What else do I need to know?**

All research is looked at by an independent group of people, called a Research Ethics Committee, to protect participant’s interests. This study has been reviewed and given favourable opinion by East Midlands - Nottingham 2 Research Ethics Committee.

So, in summary, what would happen if I decide to take part in the study?

- Your child would receive one dose of 4CMenB vaccine if they were vaccinated as an infant or toddler
- Your child would receive two doses of 4CMenB, one month or one year apart, if they have never received this vaccine before
- A member of the research team would give the vaccines at your home or appriopriate venue before or after school, at weekends or in the school holidays.
- Your child will have four blood samples taken during the study spaced out over one year
- You will need to complete an electronic diary online for 7 days after each vaccine
- You will be given and shown how to use a 24 hour temperature monitoring device after each vaccine to be worn around your child’s wrist.
- You would have 24-hour telephone access to a study doctor in case you had any concerns following vaccinations

### **What should I do now if I’m interested in taking part?**

You do not need to make a final decision straight away. If you decide to take part in this study, the next step would be to proceed to www.menbb.org.uk to complete the online eligibility and then we will contact you to arrange the first study visit if appropriate. Alternatively, you can contact the research team, who will be happy to discuss the study with you, answer any questions you have, screen your child and book the study visit over the phone. If your response reaches us after recruitment is closed we will contact you to let you know.

For those we are re-contacting, if we have not heard from you within 2 weeks we may send you a recruitment pack either through your GP or through Child Health Information Services. We may also contact you through other methods such as an email or phone call to invite your child to participate in this study.

**Contact Details:**

Oxford Vaccine Group

Centre for Clinical Vaccinology and Tropical Medicine (CCVTM )

Churchill Hospital

Oxford,

OX3 7LE

**Tel:** 01865 611400 **Email:** [info@ovg.ox.ac.uk](mailto:info@ovg.ox.ac.uk)

Thank you for considering taking part in this study.

| Yours sincerely, |  |
| --- | --- |
| **Professor Andrew Pollard**  Study Chief Investigator  Professor of Paediatric Infection and Immunity  Honorary Consultant Paediatrician | |

| 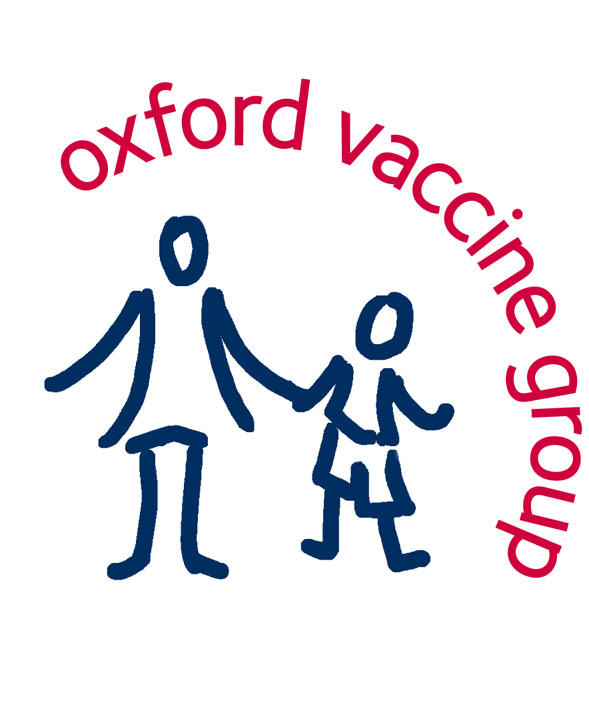 |
| --- |

OXFORD VACCINE GROUP


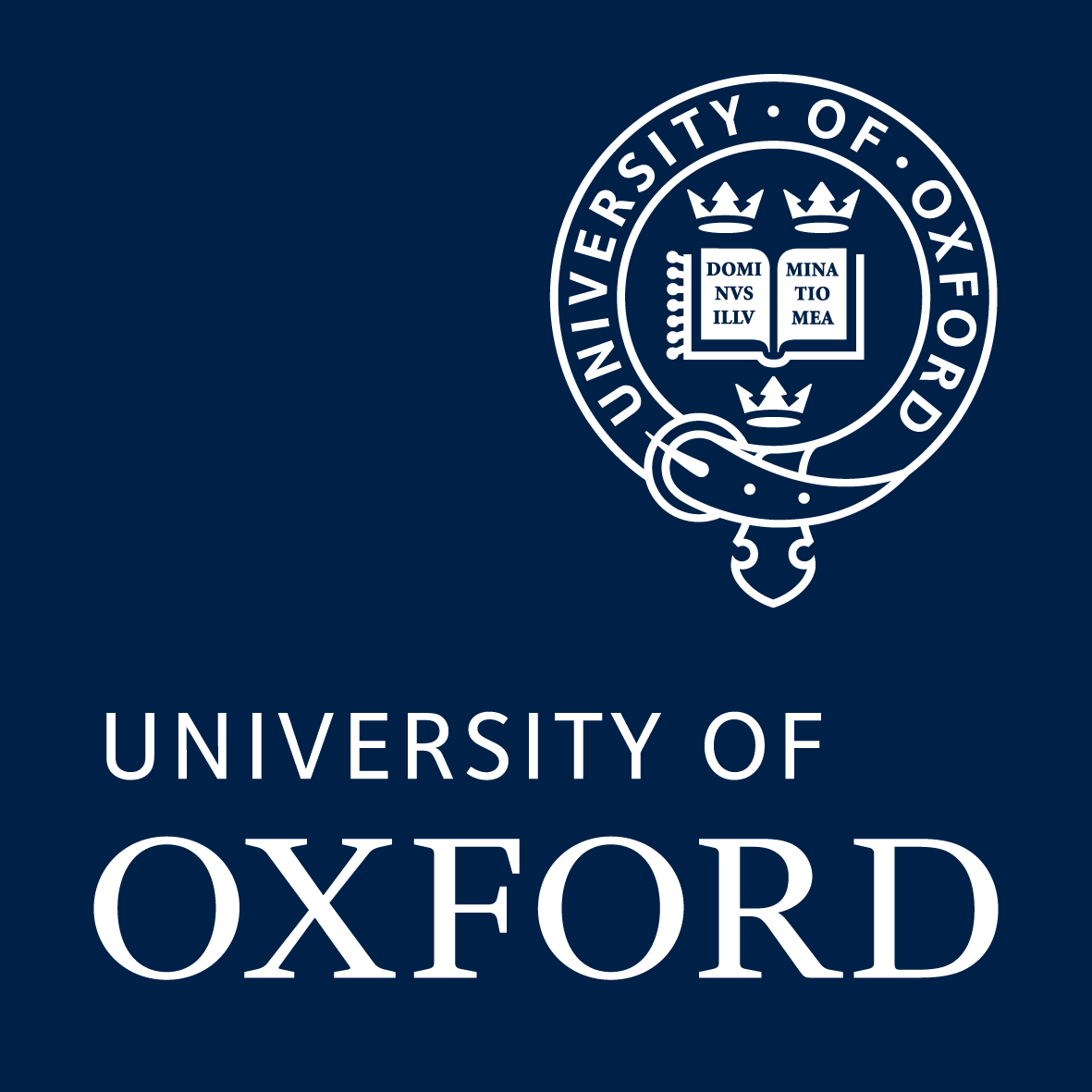


| **Meningococcal B Booster Vaccine**  **in Young People** |
| --- |

# Information booklet for young people previously vaccinated against MenB

You are invited to take part in a study looking at the responses young people make to a vaccine against a germ called meningococcus group B (MenB). MenB can cause lots of diseases, including meningitis. We want to understand how young people respond to vaccination against this germ to help to protect them in the future.

Before you decide whether you would like to take part, it is important for you to understand what’s involved. Please carefully read the information in this booklet which explains more. If you have any questions please ask your family, or a nurse or doctor at the Oxford Vaccine Group.

**Contact Details**

Oxford Vaccine Group

Centre for Clinical Vaccinology and Tropical Medicine (CCVTM)

Churchill Hospital

Oxford OX3 7LE

Tel/Fax: 01865 611400

Email: [info@ovg.ox.ac.uk](mailto:info@ovg.ox.ac.uk)

**Why am I being invited to take part in this study?**

You are being asked because you took part in an Oxford Vaccine Group study when you were a baby or toddler and were vaccinated against MenB disease.

**Why are vaccines important?**

Vaccines protect us from diseases by making our bodies produce something called antibodies in our blood. Antibodies help to fight a disease without us actually getting ill. If you come into contact with a disease which you have been vaccinated or immunised against, your body can recognise it and help fight off the disease using antiboidies before you even feel sick.

### What is MenB disease?

MenB disease is caused by an infection with meningococcal B bacteria (a type of germ). MenB disease can cause meningitis, which is an inflammation or swelling of the lining of the brain, and blood poisoning. Both meningitis and blood poisoning are very serious illnesses. There are five main groups of meningococcal bacteria, but MenB is the most common in children and young adults.

**What is this study about?**

Since September 2015 all babies in the UK are offered a vaccine to protect them against MenB disease. This vaccine is called 4CMenB. Unfortunately the protection provided by the vaccine does not last forever, because the antibodies our bodies make don’t always last for ever. Teenagers can also get MenB disease, but they are not currently offered vaccination. Therefore we think children the same age as you could benefit from having a booster dose of 4CMenB.

We hope to find out in this study if you had the 4CMenB vaccine as a baby, are you still protected from MenB disease now that you are older? Also, do you only need one further dose of the vaccine to top up (boost) your protection?

We also want to compare antibodies in children who have had the 4CMenB vaccine as babies with those that have not had it before. In order to do this, we are inviting another group of children to take part who have never had a 4CMenB vaccine.

**What do I have to do if I take part in this study?**

We will ask to see you four times in one year. We would either come to your home, or ask you to come to a place like a hospital outpatients clinic. You will receive one dose of 4CMenB vaccine, as an injection into the top of your arm.

After the vaccine we will ask you (with your parents’ help) to record how you are feeling using an eDiary on the internet. We may also ask you to wear a temperature monitoring device, about the size and shape of a small watch, for 24 hours after the vaccine was given. The watch is not waterproof however, so please take it off when you have a shower/bath or go swimming. You will have four blood tests taken over the course of a year. This will allow us to measure the antibodies in your blood, which tells us how well protected you are after being vaccinated.

The table below shows how the visits are spaced out and what would happen at each visit. You would be in group 1 to 6 (which of these groups you are in depends on when you were vaccinated as a baby).

| **Study Group** | **Visit 1** Day 0 | **Visit 2** Day 28 (one month) | **Visit 3** Day 180  (six months) | **Visit 4** Day 365 (one year) |
| --- | --- | --- | --- | --- |
| **1 to 6** had the 4CMenB vaccine as a baby | **Blood sample**  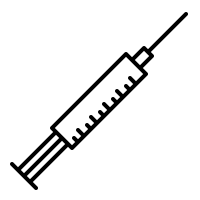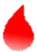 **+ Vaccine**  **Diary**  **Temperature monitoring** | 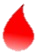**Blood sample** | 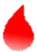**Blood sample** | 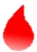**Blood sample** |
| **7**  never had 4CMenB vaccine | **Blood sample**  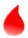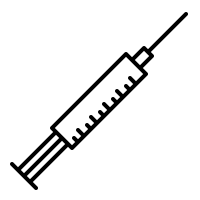**+ Vaccine**  **Diary**  **Temperature monitoring** | 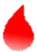**Blood sample** | 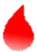**Blood sample** | **Blood sample**  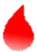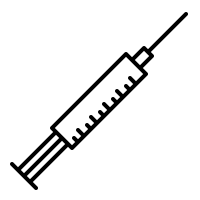**+ Vaccine**  **Diary**  **Temperature monitoring** |
| **8**  never had 4CMenB vaccine | **Blood sample**  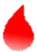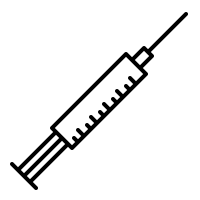**+ Vaccine**  **Diary**  **Temperature monitoring** | **Blood sample**  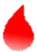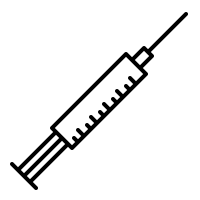**+ Vaccine**  **Diary**  **Temperature monitoring** | 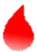**Blood sample** | **Blood sample**  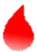 |

**Does the vaccine hurt and how may I feel after the vaccine?**

The vaccine feels like a sting. It is considered very safe as it is given to all babies. After the vaccine you may get some pain, swelling and soreness in your arm. Generally you may also feel a bit unwell and have a bit of a headache. These symptoms should disappear after one or two days and you can take some medicine to make you feel better, such as paracetamol or ibuprofen, if you need to.

**How will the blood test be taken and will they hurt?**

We will take the blood from your inner arm in the elbow crease, or the back of your hand. We will take about four teaspoons-full each time. To stop the blood test hurting we will use some numbing cream that you put on for around half an hour beforehand, or cold spray, before we take the blood. It is important that you stay still while the blood is being taken. You can distract yourself by doing something like watching television. You may get a little pain where the blood was taken from, and there may be some bruising, but this goes away after a few days.

At the end of this study, when we have finished doing our tests on your blood, we would like to share it with other researchers so they can make use of it too. This means it would be stored in something called a BioBank for other researchers; however, it will not be linked with any of your contact details.

**Who else would know I was taking part in this study?**

We would let your family doctor know that you were taking part in this study. Other than this we would not tell anyone else you were taking part. The results of this study will be written about in a scientific magazine, but you would not be able to be identified.

**Will taking part in this study help me?**

Yes. Being vaccinated with 4CMenB vaccine should protect you from MenB disease. What is learnt from this study may also help to stop other children from being unwell in the future.

**Do I have to take part in this study?**

No. Taking part in research is voluntary, which means you don’t have to agree if you don’t want to. You can also change your mind about taking part at any time: if so, please have your parents contact us.

**What else do I need to know?**

All research is looked at by an independent group of people, called a Research Ethics Committee, to protect your interests. This study has been reviewed and given favourable opinion by East Midlands Nottingham 2 Research Ethics Committee.

**What do I do now?**

You don’t have to decide straight away if you want to take part. It might be a good idea to talk to your parents before you make up your mind. You can also contact the Oxford Vaccine Group if you have any questions.

Thank you for taking the time to read this booklet!

MenB B Study Team

Thank you very much for your help with this research.

**Contact Details**

Oxford Vaccine Group

Centre for Clinical Vaccinology and Tropical Medicine (CCVTM)

Churchill Hospital

Oxford OX3 7LE

Tel/Fax: 01865 611400

Email: [info@ovg.ox.ac.uk](mailto:info@ovg.ox.ac.uk)

Website: [www.menbb.org.uk](http://www.menbb.org.uk)


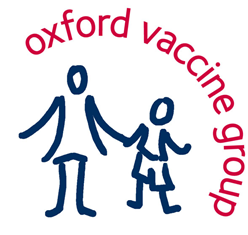


| 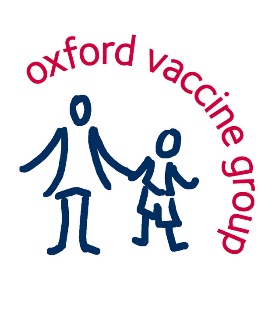 |
| --- |

OXFORD VACCINE GROUP


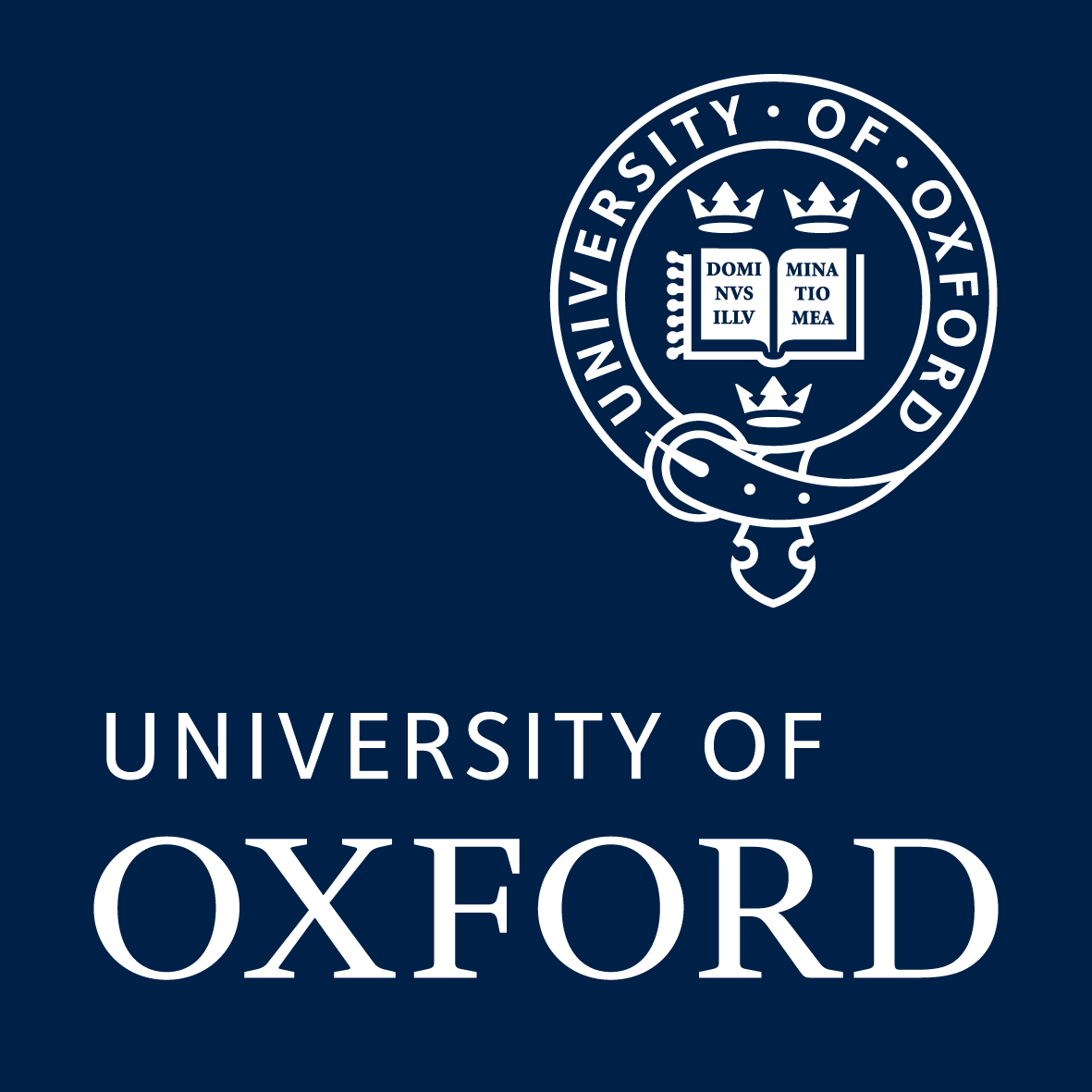


| **Meningococcal B Booster Vaccine**  **in Young People** |
| --- |

# Information booklet for young people

You are invited to take part in a study looking at the responses young people make to a vaccine against a germ called meningococcus group B (MenB). MenB can cause lots of diseases, including meningitis. We want to understand how young people respond to vaccination against this germ to help to protect them in the future.

Before you decide whether you would like to take part, it is important for you to understand what’s involved. Please carefully read the information in this booklet which explains more. If you have any questions please ask your family, or a nurse or doctor at the Oxford Vaccine Group.

**Contact Details**

Oxford Vaccine Group

Centre for Clinical Vaccinology and Tropical Medicine (CCVTM)

Churchill Hospital

Oxford OX3 7LE

Tel/Fax: 01865 611400

Email: [info@ovg.ox.ac.uk](mailto:info@ovg.ox.ac.uk)

**Why am I being invited to take part in this study?**

You are being asked because you haven’t had a MenB vaccine before, and you are the same age as a group of children who took part in an Oxford Vaccine Group study when they were a baby or toddler and were vaccinated against MenB disease.

**Why are vaccines important?**

Vaccines protect us from diseases by making our bodies produce something called antibodies in our blood. Antibodies help to fight a disease without us actually getting ill. If you come into contact with a disease which you have been vaccinated or immunised against, your body can recognise it and help fight off the disease using antiboidies before you even feel sick.

### What is MenB disease?

MenB disease is caused by an infection with meningococcal B bacteria (a type of germ). MenB disease can cause meningitis, which is an inflammation or swelling of the lining of the brain, and blood poisoning. Both meningitis and blood poisoning are very serious illnesses. There are five main groups of meningococcal bacteria, but MenB is the most common in children and young adults.

**What is this study about?**

Since September 2015 all babies in the UK are offered a vaccine to protect them against MenB disease. This vaccine is called 4CMenB. Unfortunately, the protection provided by the vaccine does not last forever because the antibodies our bodies make don’t always last for ever. Teenagers can also get MenB disease, but they are not currently offered vaccination.

In this study, we want to compare antibodies in children who have had the 4CMenB vaccine as babies with those that have not had it before. In order to do this, we are inviting two groups of children to take part. There will be a group who have never had the vaccine before (you will be in this group if you choose to take part) and another group who have had the vaccine as babies as part of another research study.

**What do I have to do if I take part in this study?**

We will ask to see you four times in one year. We would either come to your home, or ask you to come to a place like a hospital outpatients clinic. You will receive two doses of 4CMenB vaccine, as an injection into the top of your arm. The second dose will either be given one month or one year after the first, depending on which group you are put in; group 7 or 8. Deciding which group you are in is done by chance, a bit like flipping a coin. It is not up to you, your parents or us. There is equal chance of you being in group 7 or group 8.

After each dose of 4CMenB vaccine we will ask you (with your parents’ help) to record how you are feeling using an eDiary on the internet. We may also ask you to wear a temperature monitoring device, about the size and shape of a small watch, for 24 hours after the vaccine was given. As this temperature watch is not waterproof, please take it off when you have a bath/shower or if you go swimming. You will have four blood tests taken over the course of a year. This will allow us to measure the antibodies in your blood, which tells us how well protected you are after being vaccinated.

The table below shows how the visits are spaced out and what would happen at each visit. You would be in group 7 or 8. Whichever group you are in, you will receive two injections of the vaccine, this is because one dose is not enough to give you any protection. However, group 7 have their second injection later than group 8 so we can compare between the different groups more easily.

| **Study Group** | **Visit 1** Day 0 | **Visit 2** Day 28 (one month) | **Visit** 3 Day 180  (six months) | **Visit 4** Day 365 (one year) |
| --- | --- | --- | --- | --- |
| **1 to 6** had the 4CMenB vaccine as a baby | **Blood sample**  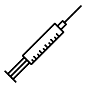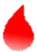 **+ Vaccine**  **Diary**  **Temperature monitoring** | 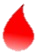**Blood sample** | 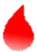**Blood sample** | 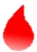**Blood sample** |
| **7**  never had 4CMenB vaccine | **Blood sample**  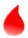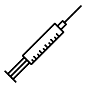**+ Vaccine**  **Diary**  **Temperature monitoring** | 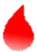**Blood sample** | 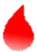**Blood sample** | **Blood sample**  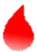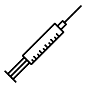**+ Vaccine**  **Diary**  **Temperature monitoring** |
| **8**  never had 4CMenB vaccine | **Blood sample**  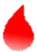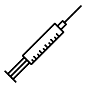**+ Vaccine**  **Diary**  **Temperature monitoring** | **Blood sample**  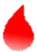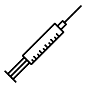**+ Vaccine**  **Diary**  **Temperature monitoring** | 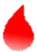**Blood sample** | **Blood sample**  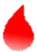 |

**Does the vaccine hurt and how may I feel after the vaccine?**

The vaccine feels like a sting. It is considered very safe as it is given to all babies. After the vaccine you may get some pain, swelling and soreness in your arm. Generally you may also feel a bit unwell and have a bit of a headache. These symptoms should disappear after one or two days and you can take some medicine to make you feel better, such as paracetamol or ibuprofen, if you need to.

**How will the blood test be taken and will they hurt?**

We will take the blood from your inner arm in the elbow crease, or the back of your hand. We will take about four teaspoons-full each time. To stop the blood test hurting we will use some numbing cream that you put on for around half an hour beforehand, or cold spray, before we take the blood. It is important that you stay still while the blood is being taken. You can distract yourself by doing something like watching television. You may get a little pain where the blood was taken from, and there may be some bruising, but this goes away after a few days.

At the end of this study, when we have finished doing our tests on your blood, we would like to share it with other researchers so they can make use of it too. This means it would be stored in something called a BioBank for other researchers; however, it will not be linked with any of your contact details.

**Who else would know I was taking part in this study?**

We would let your family doctor know that you were taking part in this study. Other than this we would not tell anyone else you were taking part. The results of this study will be written about in a scientific magazine, but you would not be able to be identified.

**Will taking part in this study help me?**

Yes. Being vaccinated with 4CMenB vaccine should protect you from MenB disease. What is learnt from this study may also help to stop other children from being unwell in the future.

**Do I have to take part in this study?**

No. Taking part in research is voluntary, which means you don’t have to agree if you don’t want to. You can also change your mind about taking part at any time: if so, please have your parents contact us.

**What else do I need to know?**

All research is looked at by an independent group of people, called a Research Ethics Committee, to protect your interests. This study has been reviewed and given favourable opinion by East Midlands Nottingham 2 Research Ethics Committee.

**What do I do now?**

You don’t have to decide straight away if you want to take part. It might be a good idea to talk to your parents before you make up your mind. You can also contact the Oxford Vaccine Group if you have any questions.

Thank you for taking the time to read this booklet!

MenBB Study Team

Thank you very much for your help with this research.

**Contact Details**

Oxford Vaccine Group

Centre for Clinical Vaccinology and Tropical Medicine (CCVTM)

Churchill Hospital

Oxford OX3 7LE

Tel/Fax: 01865 611400

Email: [info@ovg.ox.ac.uk](mailto:info@ovg.ox.ac.uk)

Website: [www.menbb.org.uk](http://www.menbb.org.uk)


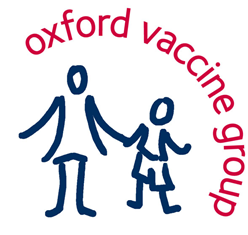


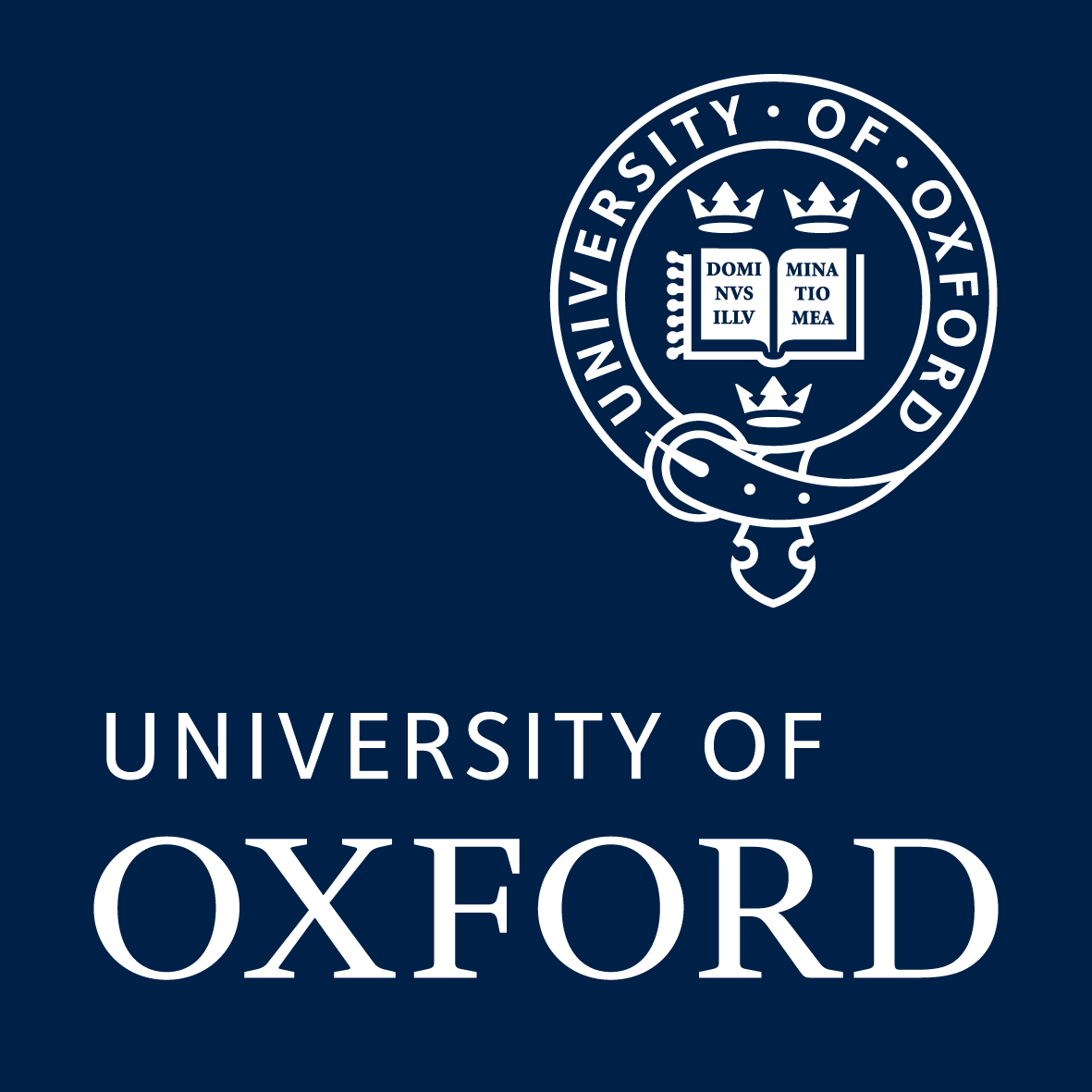


Oxford Vaccine Group

University of Oxford

Centre for Clinical Vaccinology and Tropical Medicine,

Churchill Hospital, Headington, Oxford OX3 7LE

Tel: 01865 611400 info@ovg.ox.ac.uk www.ovg.ox.ac.uk


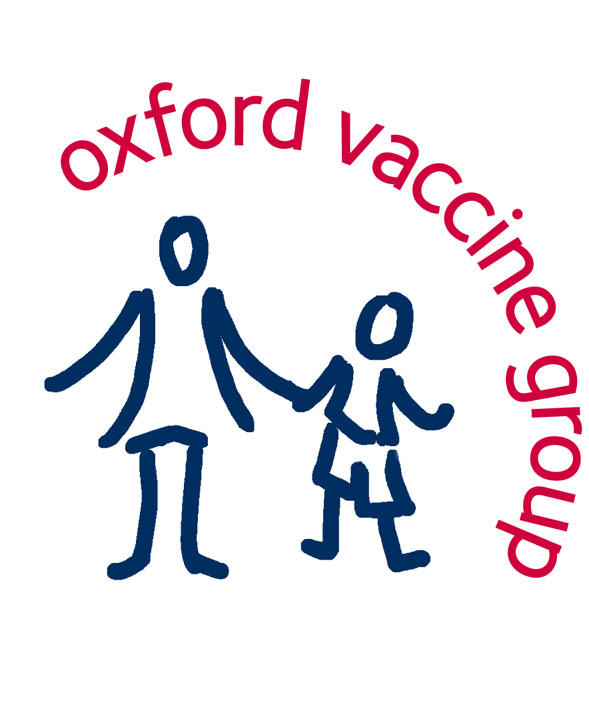


**Meningococcal B Booster Vaccine in Young People**

**Informed Consent Form**

**Chief Investigator: Professor Pollard IRAS ID: 231165**

Name of Participant: …………………….. Participant initials: |___| |___|

*Please* ***initial*** *each section*

| - I confirm that I have read the information booklet ‘Meningococcal B Booster Vaccine in Young People, (version 2.0, dated 19-March-2018). I have had the opportunity to consider the information, discuss the study, to ask questions and have had these answered satisfactorily. | |  | |
| --- | --- | --- | --- |
| - I understand that my child’s participation in this study is voluntary and that I am free to withdraw at any time, without needing to give a reason and that withdrawal does not affect their medical care or any legal rights. | |  | |
| - I agree to my child’s GP being informed about their participation in this study, and understand that additional information regarding their medical history may be sought from them and/or other treating doctors. | |  | |
| - I understand and give permission for relevant sections of my child’s medical notes and data already in existence and/or collected during the study to be looked at, if necessary, by authorised individuals from the study team, representatives of the University of Oxford, or by persons from regulatory authorities where it is relevant to my child taking part in this research. I give permission for these individuals to have access to my records. | |  | |
| - I agree to the researchers storing and sharing my child’s personal information as described in the information booklet. | |  | |
| - I agree to appropriately trained members of study staff taking and storing blood samples from my child as explained in the information booklet. | |  | |
| - I consider all samples provided by my child as a gift to the University of Oxford and I understand neither my child nor I will not gain any direct personal or financial benefit from providing them or from my child taking part in the study. | |  | |
| - I agree to my child’s sample/s being shipped outside the United Kingdom, including outside of the European Union for analysis as part of this study. | |  | |
| - I understand that the information collected about my child may be used in an anonymous form to support other research in the future. It will not be possible for my child to be identified by it. | |  | |
| If all of the sentences above are initialled, meaning “yes”, then please continue: | | | |
| - I voluntarily agree for my child to take part in this study |  | | |
| ***Optional*** *(please note that you can still participate in this study whether or not you agree to the next statements):* | | | |
| - I agree to be contacted about ethically approved research studies for which my child may be suitable. I understand that agreeing to be contacted does not oblige my child to participate in any further studies. | Yes | | No |
|  |  | |  |
| - I agree to being contacted about studies in the future that are related to this study and I understand that I would be under no obligation to take part in these future | Yes | | No |
|  |  | |  |

Name: ………………………………..Relationship to child:……………………

Signature:………………………………………….Date: |__ __|__ __ __|20__ __|

Name of person receiving consent:……………………………………………………

Signature: …………………………………… Date: |__ __|__ __ __|20__ __|

*1 copy for participant; 1 copy for researcher site file (original);*


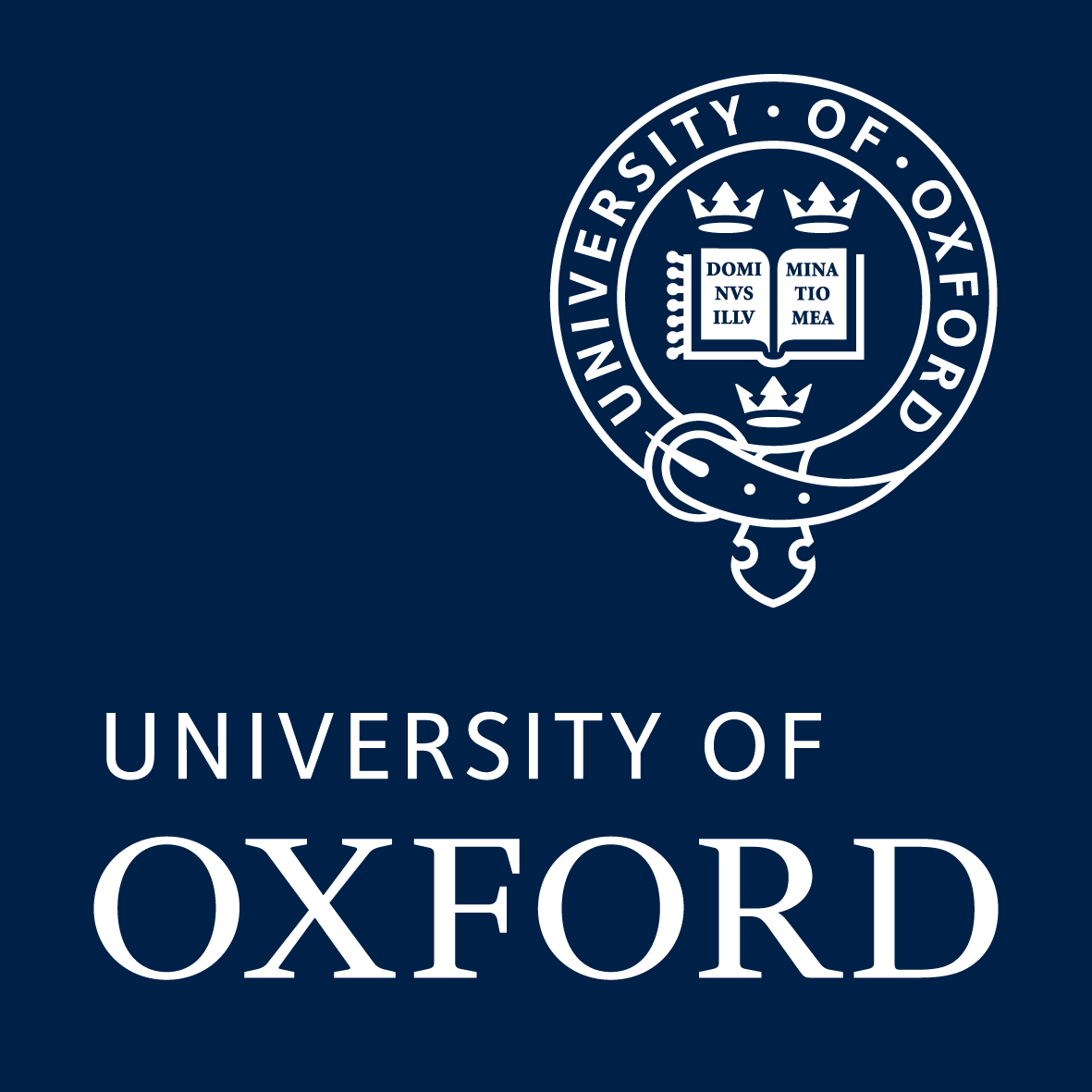


Oxford Vaccine Group

University of Oxford

Centre for Clinical Vaccinology and Tropical Medicine,

Churchill Hospital, Headington, Oxford OX3 7LE

Tel: 01865 611400 info@ovg.ox.ac.uk www.ovg.ox.ac.uk


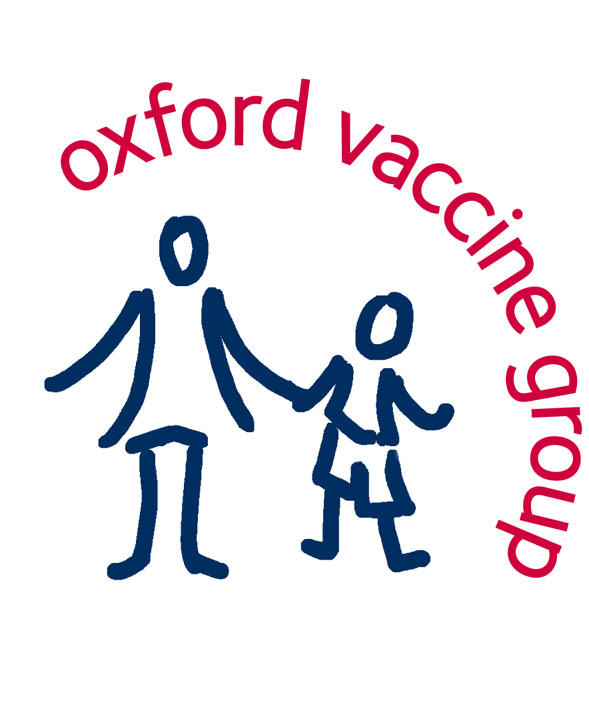


**Meningococcal B Booster Vaccine in Young People**

**Informed Assent Form**

**Chief Investigator: Professor Pollard IRAS ID: 231165**

Name of Participant: ………………………. Participant initials: |___| |___|

*Please* ***circle*** *yes* ***or*** *no for* *each section*

| 1. Have you read about this research study? | Yes | No |
| --- | --- | --- |
| 1. Do you understand what this research study is about? | Yes | No |
| 1. Have you asked any questions you want to? | Yes | No |
| 1. Have you had time to think about taking part? | Yes | No |
| 1. Do you know that you are allowed to change your mind about taking part at any time? | Yes | No |
| 1. Are you happy to take part? | Yes | No |

Your Name: ………………………………………………………

Your Signature:………………………………Date: |__ __|__ __ __|20__ __|

Parent name: ………………………………………………………

Parent signature:………………………………Date: |__ __|__ __ __|20__ __|

Name of study staff: ……………………………………………………………

Signature: …………………………………… Date: |__ __|__ __ __|20__ __|

*1 copy for participant; 1 copy for researcher site file (original);*

**33) Biological specimens:**


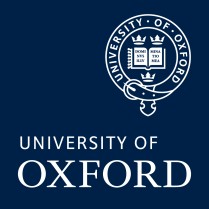


| 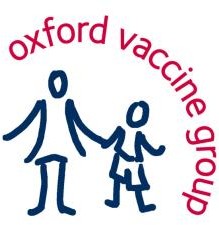 | Oxford Vaccine Group  University of Oxford  Centre for Clinical Vaccinology and Tropical Medicine,  Churchill Hospital, Headington, Oxford OX3 7LE  Tel: 01865 611400 [info@ovg.ox.ac.uk](mailto:info@ovg.ox.ac.uk) |
| --- | --- |

OXFORD VACCINE CENTRE

**Oxford Vaccine Centre Biobank**

**Assenting information booklet for Participants aged 11 to 15 years**

We would like to thank you for being involved in a study being run by the Oxford Vaccine Centre. We are now asking if you would be happy for us to store the samples from this study in a ‘Biobank’. This is voluntary, which means that you can decide whether or not you want to give us permission to do this.

## What is a Biobank?

You are receiving this booklet because you are involved with an Oxford Vaccine Group study in which we are taking samples such as blood tests or throat/nose swabs. With your permission, we would like to use some of these samples to answer more questions about how people your age are protected against infections. This would involve us storing some of your samples in something called a ‘Biobank’ after the original study has ended. This is like a library, but stores study samples instead of books! This Biobank has approval from the people that make sure that research studies are done properly (an ethics committee).

## What would I need to do?

If you take part it would NOT involve any extra visits or needles to take blood over what you are already doing. All children participating in your Oxford Vaccine Centre study are being invited to allow us to store their samples in the Biobank.

What happens to the samples in the Blobank?

If you agree to us storing your samples in the Biobank, we label them with a study number, but not your name. Some of the ways we might use the Biobank samples are:

- getting a sample of your genes. This is the DNA ‘code’ in your body that makes your body the way it is. This would help us study how each person’s genetic code affects how well they respond to vaccines
- studying your blood sample to look at the level of protection against common childhood diseases.
- other tests to help us learn about how children’s bodies fight off infections.

## Who else would know I was taking part in this extra study

We may need to tell your family doctor to find out which vaccines you have already had. Other than this we would not tell anyone else you were taking part in the Biobank – your involvement would be confidential.

## Will taking part in the Biobank help me?

Taking part in the Biobank would not help you directly but it might help us stop other children getting sick in the future.

## What happens if I say ‘no’?

Taking part in research is voluntary, which means you don’t have to agree if you don’t want to. If you decide not to take part in the Biobank, you can still take part in the study you are already involved in, and it wouldn’t affect the way your doctor or other people take care of you. You can also change your mind about taking part in the biobank at any time: if so, please have your parents contact us. When you are older, you will be able to do this on your own behalf and you don’t have to give us a reason why.

What do I do now?

You don’t have to decide straight away if you want to take part. It might be a good idea if you talk to your parents before you make up your mind. When we next see you we will be asking you if you are happy to take part in the Biobank or not and will be happy to answer any questions you might have. Thank you for taking the time to read this booklet!

| 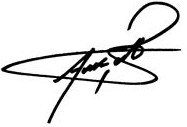 | 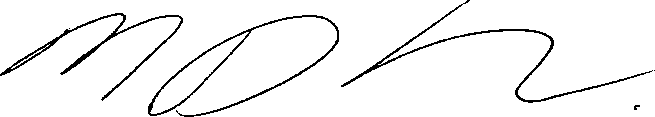 | 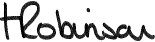 |
| --- | --- | --- |
| Professor Andrew Pollard Study Chief Investigator Professor of Paediatric Infection and Immunity Honorary Consultant  Paediatrician | Dr. Matthew Snape Consultant in Paediatrics and Vaccinology | Hannah Robinson Senior Research Nurse |

**Oxford Vaccine Centre Biobank**

**ASSENT FORM**

*(Appropriate for 11 to 15 year olds)*

Please circle

one answer

| Have you read about this research study? | Yes / No |
| --- | --- |
| Do you understand what the research study is about? | Yes / No |
| Have you asked any questions you want to? | Yes / No |
| Have you had time to think about taking part? | Yes / No |
| Are you happy for your medical notes to be looked at by the study team to see what vaccines you have had? | Yes / No |
| Are you happy to take part? | Yes / No |

# If you don’t want to take part, please let your doctor know.

# If you do want to take part, please write below:

| Your Name | **Your Signature** | Date |
| --- | --- | --- |

_________________________________________________________________________________

Thank you very much for your help with this research.


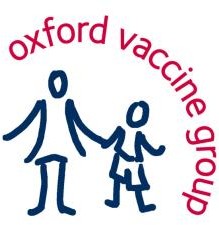

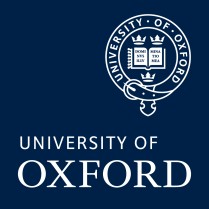


OXFORD VACCINE CENTRE

**Oxford Vaccine Centre Biobank**

# Information Booklet

We would like to thank you for your interest/involvement in an Oxford Vaccine Centre (OVC) research study. We are writing now to see if you would be willing for a small amount of the samples taken in this study to be stored in the Oxford Vaccine Centre Biobank.

This is completely voluntary. Before you make your decision, it is important for you to understand what participation of your child would involve. Please take some time to read the information in this booklet carefully and discuss with others if you wish.

If anything is unclear or you would like further information please feel free to contact a member of the Oxford Vaccine Centre team:

Centre for Clinical Vaccinology and Tropical Medicine Churchill Hospital, Oxford, OX3 7LE

Tel/Fax: 01865 611400

Email: [info@ovg.ox.ac.uk](mailto:ovg@paediatrics.ox.ac.uk)

Thank you for taking the time to consider contributing to the Oxford Vaccine Centre Biobank.

## What is Oxford Vaccine Centre Biobank?

You are receiving this information booklet as your child is currently taking part in an Oxford Vaccine Centre research study. In this study samples of blood or other biological materials are being taken, and with your permission, we would like to:

- obtain a sample of your child’s genetic material

and/or

- store any biological samples such as whole blood (with all the blood cells still present), throat or nose swabs, stool samples or biopsy samples

and/or

- store serum after the study your child is in has ended. (Serum is the liquid that remains after all blood cells have been removed from blood.)

These samples would be stored in the Oxford Vaccine Centre Biobank.

By collecting genetic samples from thousands of participants across different studies, we hope to understand how genes influence the immune response to immunisation and infectious diseases. Storing biological samples or serum would help us with future research into infection and immunity, for example by helping us assess the level of immunity against an infectious disease within the wider population. This information may in turn help in the understanding of diseases and the development of newer, better vaccines.

Why is my child being invited to contribute to the Oxford Vaccine Centre Biobank?

Your child is being invited to contribute to the Biobank as they are currently taking part in another study being conducted by the Oxford Vaccine Centre, or are considering doing so. Contributing to the Biobank is entirely voluntary, and your decision about whether or not you want your child to do this will not affect your child’s involvement in any other study.

What would this involve?

If you agreed to samples from your child being stored in the Oxford Vaccine Centre Biobank it would NOT involve any extra procedures. Instead, these would be obtained from the remainder of the samples obtained during your child’s study. With your permission, we may also contact your GP or NHS immunisation databases to review the vaccines your child has previously received. All parents/guardians of children participating in this study conducted at the Oxford Vaccine Centre are being invited to participate in this additional research.

## What are the benefits and risks of taking part in the Biobank?

Taking part in the Biobank would not have any direct benefit to you or your child. The genetic testing would look for naturally occurring variation in the genetic code. You would not be informed of the results of analyses conducted on the samples in the Biobank. Although we may be obtaining details of the vaccines your child has received, we would not be taking on the responsibility for making sure these are ‘up to date’. As there are no procedures beyond those required for your study, there is no additional risk in participating in the Biobank.

## Who will have access to the information obtained in the Biobank?

Any records with your child’s information would be held by the Oxford Vaccine Centre or designated archiving facilities. Your child’s participation in the Biobank would remain confidential; however we may need to inform your GP about their involvement if we need to obtain information about the vaccines they have previously received. In order to ensure that the Biobank was being managed correctly, for monitoring and audit purposes your child’s records may be read (but not kept) by staff of the Clinical Trials and Research Governance Office, University of Oxford. Other relevant regulatory bodies would also have access to such records (such as the ethics committee and human tissue authority).

## What would happen to the samples stored in the Biobank?

Any samples stored in the Biobank would be labeled with a Biobank number, but not your child’s name. Those testing the samples would therefore not know the name or other personal details of the people providing the samples. Following testing, your child’s samples would continue to be stored in the Oxford Vaccine Centre Biobank for future research related to infection and immunity.

Although those doing the testing and analysis would not be able to identify which individual a sample had come from, should this be necessary at any point (e.g. if you or your child changed your mind and want your samples removed from the Biobank) information linking each sample to the individual providing it, would be stored securely by the Oxford Vaccine Group Biobank designated individual who is independent from the researchers using the OVC Biobank samples. Access to the ‘linking’ data would only be allowed with appropriate approvals. Also, if you informed us that your child had previously had samples taken in another Oxford Vaccine Centre study, with your permission we would temporarily re-establish the connection between stored Biobank samples and your child’s name to allow us to ‘link-up’ all samples provided by you in different studies.

Testing on the samples stored in the Biobank would be performed either in Oxford or at other laboratories with specific expertise; these may be outside of the European Union. Research may be performed in collaboration with external research groups, including pharmaceutical companies, but samples would not be used directly in animal research.

## What else do I need to know?

Consent for having your child’s samples stored in the Biobank is voluntary, and if you do not agree this would not affect your participation in the study in which the samples were obtained, nor your child’s routine care. When your child reaches the age of sixteen, they will have the right to have their samples withdrawn from the study. Prior to this you will have the right. In either case, you or your child would need to contact OVC themselves if they wished to do this. The Oxford Vaccine Centre Biobank has the approval of the HRA South Central – Hampshire B Research Ethics Committee. If you wish to complain about any aspect of the way in which you or your child have been approached or treated during the course of this study, we suggest that you contact us, or alternatively the University of Oxford Clinical Trials and Research Governance office on 01865 572224.

## What do I do now?

Thank you for considering taking part in the Oxford Vaccine Centre Biobank. If you wish to discuss the Biobank in more detail, please contact us by telephone (01865 611400) or e-mail ([info@ovg.ox.ac.uk](mailto:info@ovg.ox.ac.uk)). Otherwise we will ask whether you are willing for your child to contribute to the Oxford Vaccine Centre Biobank at a study visit.


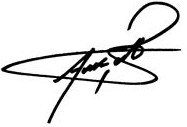
Yours sincerely,

|  | 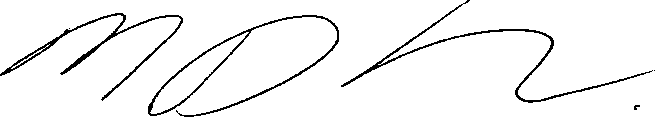 | 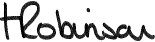 |
| --- | --- | --- |
| Professor Andrew Pollard Study Chief Investigator Professor of Paediatric Infection and Immunity Honorary Consultant  Paediatrician | Dr. Matthew Snape Consultant in Paediatrics and Vaccinology | Hannah Robinson Senior Research Nurse |


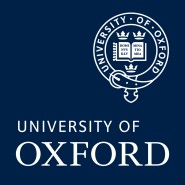


| 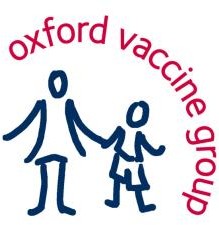 | Oxford Vaccine Group  University of Oxford  Centre for Clinical Vaccinology and Tropical Medicine,  Churchill Hospital, Headington, Oxford OX3 7LE  Tel: 01865 611400 [info@ovg.ox.ac.uk](mailto:info@ovg.ox.ac.uk) |
| --- | --- |

**Oxford Vaccine Centre Biobank**

# Informed Consent Form

Name of Participant: Participant Initials: |____| |____|

*Please initial each section if you agree with the statement*

| I have read the Oxford Vaccine Centre Child Information Booklet  and Consent (Version 8.4, dated 09 June 2017). | \|__ __\| |
| --- | --- |
| My child has had the nature of their participation in the Biobank study explained to them (to be used for children aged 6 to 15). | \|__ __\| |
| I have had the opportunity to ask questions about the Biobank and had them answered. | \|__ __\| |
| I understand that I am free to refuse my child’s participation in the Biobank without affecting their medical care. | \|__ __\| |
| When my child reaches the age of sixteen, they will have the right to have their samples withdrawn from the study. Prior to this I will have this right. In either case, my child or myself would need to contact OVC themselves if they wished to do this. | \|__ __\| |
| I am aware that the samples provided and anonymised data may be sent outside of the EU and shared with commercial research collaborators. | \|__ __\| |
| I consider these samples a gift to the University of Oxford and I understand I and my child will not gain any direct personal benefit from this. | \|__ __\| |
| I agree to take part in this research study. | \|__ __\| |

*If all above are initialled, meaning “yes”, then please place your initials in the appropriate boxes below:*

| I agree to you contacting my GP or NHS immunisation databases to find out which vaccines my child has previously received. | YES | NO |  |
| --- | --- | --- | --- |
| I agree that genetic material from my child’s study samples may be stored and used in future vaccine related research.  *(Please initial ‘N/A’ if the study team member indicates no genes will be stored)* | YES | NO | N/A |
| I agree that biological samples from my child’s study samples may be stored and used in future vaccine related research.  *(Please initial ‘N/A’ if the study team member indicates no biological samples will be stored)* | YES | NO | N/A |
| I agree that my child’s blood serum may be stored and used in future research.  *(Please initial ‘N/A’ if the study team member indicates no serum will be stored)* | YES | NO | N/A |
| My child has previously provided samples in an Oxford Vaccine Centre study and I agree to staff temporarily re-establishing the connection between stored samples and my child’s name so that samples can be linked.  *(Please initial ‘N/A’ if your child has not previously provided samples in an Oxford Vaccine Centre study or if you do not know whether this is the case)* | YES | NO | N/A |
|  |  |  |  |
|  |  |  |  |

Name: ………………………………………………………………………………………………………………………………..

Relationship to Child (parent or legal guardian only): …………………………………………………………

Signature: ……………………………………………………….. Date: |____| |____| |___ ___|

Study staff name: ………………………………………………………………………………………………………........

Signature: ……………………………………………………….. Date: |____| |____| |___ ___|

**References:**

1. Laboratory confirmed cases of invasive meningococcal infection (England): January to March 2018. Public Health England; 2018 22 June 2018.

2. Bruge J, Bouveret-Le Cam N, Danve B, Rougon G, Schulz D. Clinical evaluation of a group B meningococcal N-propionylated polysaccharide conjugate vaccine in adult, male volunteers. Vaccine. 2004;22(9):1087-96.

3. Joint Committee on Vaccination and Immunisation Minute of the meeting on Tuesday 11 and Wednesday 12 February 2014 [Internet]. 2014. Available from: <https://app.box.com/s/iddfb4ppwkmtjusir2tc/file/229171703722>.

4. Banzhoff A. Multicomponent meningococcal B vaccination (4CMenB) of adolescents and college students in the United States. Ther Adv Vaccines. 2017;5(1):3-14.

5. Pelton SI. The Global Evolution of Meningococcal Epidemiology Following the Introduction of Meningococcal Vaccines. Journal of Adolescent Health. 2016;59(2, Supplement):S3-S11.

6. Findlow J, Borrow R, Snape MD, Dawson T, Holland A, John TM, et al. Multicenter, open-label, randomized phase II controlled trial of an investigational recombinant Meningococcal serogroup B vaccine with and without outer membrane vesicles, administered in infancy. Clin Infect Dis. 2010;51(10):1127-37.

7. Snape MD, Saroey P, John TM, Robinson H, Kelly S, Gossger N, et al. Persistence of bactericidal antibodies following early infant vaccination with a serogroup B meningococcal vaccine and immunogenicity of a preschool booster dose. CMAJ. 2013;185(15):E715-24.

8. Perrett KP, McVernon J, Richmond PC, Marshall H, Nissen M, August A, et al. Immune responses to a recombinant, four-component, meningococcal serogroup B vaccine (4CMenB) in adolescents: a phase III, randomized, multicentre, lot-to-lot consistency study. Vaccine. 2015;33(39):5217-24.

9. McNamara LA, Shumate AM, Johnsen P, MacNeil JR, Patel M, Bhavsar T, et al. First Use of a Serogroup B Meningococcal Vaccine in the US in Response to a University Outbreak. Pediatrics. 2015;135(5):798-804.

10. Santolaya ME, O'Ryan ML, Valenzuela MT, Prado V, Vergara R, Munoz A, et al. Immunogenicity and tolerability of a multicomponent meningococcal serogroup B (4CMenB) vaccine in healthy adolescents in Chile: a phase 2b/3 randomised, observer-blind, placebo-controlled study. Lancet (London, England). 2012;379(9816):617-24.

11. Rollier CS, Dold C, Marsay L, Sadarangani M, Pollard AJ. The capsular group B meningococcal vaccine, 4CMenB : clinical experience and potential efficacy. Expert Opin Biol Ther. 2015;15(1):131-42.

12. Costa I, Pajon R, Granoff DM. Human factor H (FH) impairs protective meningococcal anti-FHbp antibody responses and the antibodies enhance FH binding. mBio. 2014;5(5):e01625-14.

13. Preventing meningitis in young people after infant immunisation: effect of a single meningococcal 4CMenB vaccine booster over 10 years of age. . [Study protocol]. In press 2018.
